# Supplementary material for: Inhibition of Resistance-Refractory P. falciparum Kinase PKG Delivers Prophylactic, Blood Stage, and Transmission-Blocking Antiplasmodial Activity
Source: Cell Chem Biol. 2020 Jul 16;27(7):806–816.e8. doi: 10.1016/j.chembiol.2020.04.001 (PMC7369637; doi:10.1016/j.chembiol.2020.04.001)
Supplement: Document S1. Figures S1–S3 [file mmc1.pdf]

Cell Chemical Biology, Volume *27*

Supplemental Information

Inhibition of Resistance-Refractory *P. falciparum* Kinase PKG Delivers Prophylactic, Blood Stage, and Transmission-Blocking Antiplasmodial Activity

Manu Vanaerschot, James M. Murithi, Charisse Flerida A. Pasaje, Sonja Ghidelli-Disse, Louis Dwomoh, Megan Bird, Natasha Spottiswoode, Nimisha Mittal, Lauren B. Arendse, Edward S. Owen, Kathryn J. Wicht, Giulia Siciliano, Markus Bösche, Tomas Yeo, T.R. Santha Kumar, Sachel Mok, Emma F. Carpenter, Marla J. Giddins, Olalla Sanz, Sabine Ottilie, Pietro Alano, Kelly Chibale, Manuel Llinás, Anne-Catrin Uhlemann, Michael Delves, Andrew B. Tobin, Christian Doerig, Elizabeth A. Winzeler, Marcus C.S. Lee, Jacquin C. Niles, and David A. Fidock

Inhibition of the resistance-refractory *P. falciparum* kinase PKG delivers prophylactic, blood stage and transmission-blocking antiplasmodial activity

Manu Vanaerschot, James M. Murithi, Charisse F.A. Pasaje, Sonja Ghidelli-Disse, Louis Dwomoh, Megan Bird, Natasha Spottiswoode, Nimisha Mittal, Lauren B. Arendse, Edward S. Owen, Kathryn J. Wicht, Giulia Siciliano, Markus Bösche, Tomas Yeo, T.R. Santha Kumar, Sachel Mok, Emma F. Carpenter, Marla J. Giddins, Olalla Sanz, Sabine Ottilie, Pietro Alano, Kelly Chibale, Manuel Llinás, Anne-Catrin Uhlemann, Michael Delves, Andrew B. Tobin, Christian Doerig, Elizabeth A. Winzeler, Marcus C.S. Lee, Jacquin C. Niles, David A. Fidock

Supplementary Figures and Tables (Table S1 is available as a separate multi-tab Excel file)

**Figure S1.**	Structure of the MMV030084 analogs tested in this study.	**p. 2**
**Figure S2.**	Female gamete formation assay results, showing little to no activity against female gametes.	**p. 3**
**Figure S3.**	Immunoblotting confirmed knock-downs of PKG, CDPK1, PP1 and URP.	**p. 4**
**Figure S4.**	Phosphorimage of recombinant TKL3 kinase inhibition studies indicate that MMV030084 and the tested analogs do not directly inhibit TKL3 activity.	**p. 5**
**Table S1 (A).**	MMV030084 asexual blood stage activity data.	
**Table S1 (B).**	Activity (IC_{50} in nM) of MMV030084 and its analogs against Dd2-B2 and a TKL3-mutant line.	
**Table S1 (C).**	Log_2 fold changes in metabolite levels induced by MMV030084 treatment of trophozoites and schizonts.	
**Table S1 (D1,2).**	Phosphorylated peptide levels in MMV030084-treated vs untreated (control) Dd2-B2 (D1) and TKL-3 KO (D2) parasites.	
**Table S1 (E1-3).**	Results from the chemoproteomic experiments.	
**Table S1 (F).**	Effect of cKDs on parasite growth.	
**Table S1 (G).**	Susceptibility data for the cKD lines to MMV030084 and MMV030734.	
**Table S1 (H)**.	MMV030084 and its analogs dock well into the crystal structure of PKG.	
**Table S1 (I).**	IC_{50} data for MMV030084-resistant lines.	
**Table S1 (J).**	Sequences of primers used to generate and validate cKD lines.	
**Table S1 (K).**	Homology regions and sgRNA fragments used to generate cKD lines.	

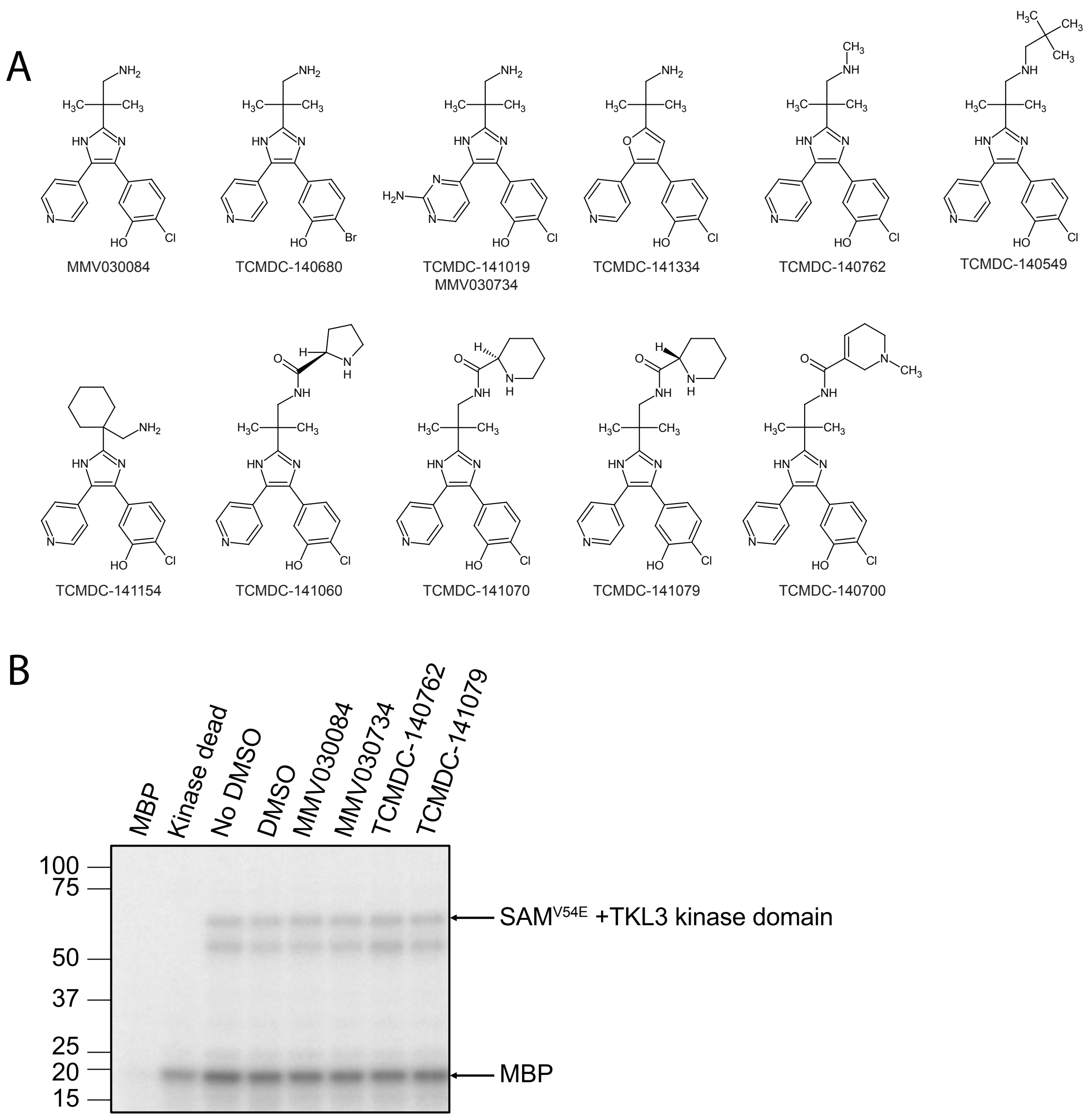

Figure S1. Structure of MMV030084 and its analogs and evidence of their lack of direct inhibition of TKL3 kinase activity (related to Figure 1 and Figure 3). (A) Analogs of MMV0300084. (B) Phosphorimage of recombinant TKL3 kinase inhibition studies indicate that MMV030084 and the tested analogs do not directly inhibit TKL3 activity. MBP: myelin basic product, SAM: sterile alpha motif.

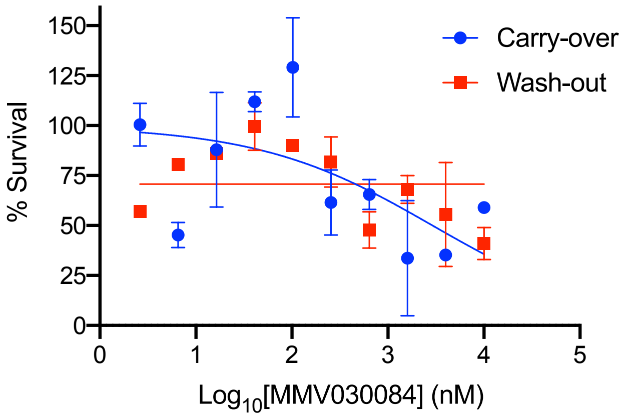

Figure S2. Female gamete formation assay results, showing little to no activity against female gametes (related to Figure 1). Error bars represent the standard error of the mean based on two independently repeated experiments.

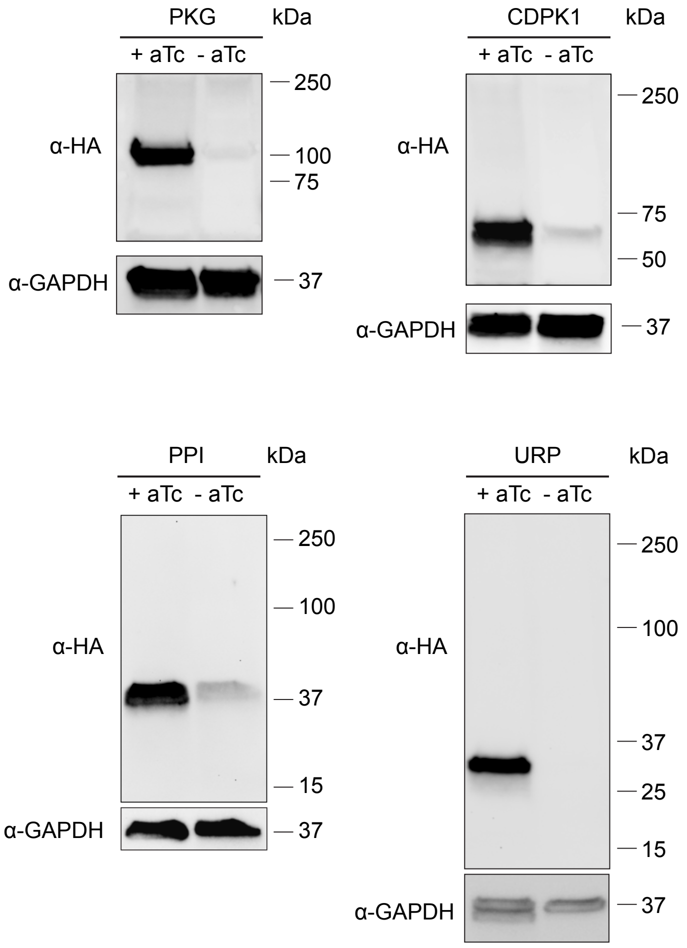

Figure S3. Immunoblotting confirmed knock-downs of PKG, CDPK1, PP1 and URP (related to Figure 3; Table S6). Proteins were probed using anti-HA antibodies, which target the HA tag that was introduced at the 3’ end of the genes together with the cKD machinery**.**
