# Supplementary material for: Inhibition of Resistance-Refractory P. falciparum Kinase PKG Delivers Prophylactic, Blood Stage, and Transmission-Blocking Antiplasmodial Activity
Source: Cell Chem Biol. 2020 Jul 16;27(7):806–816.e8. doi: 10.1016/j.chembiol.2020.04.001 (PMC7369637; doi:10.1016/j.chembiol.2020.04.001)
Supplement: Document S2. Article plus Supplemental Information [file mmc3.pdf]

Article

Cell Chemical Biology

Inhibition of Resistance-Refractory *P. falciparum* Kinase PKG Delivers Prophylactic, Blood Stage, and Transmission-Blocking Antiplasmodial Activity

Graphical Abstract

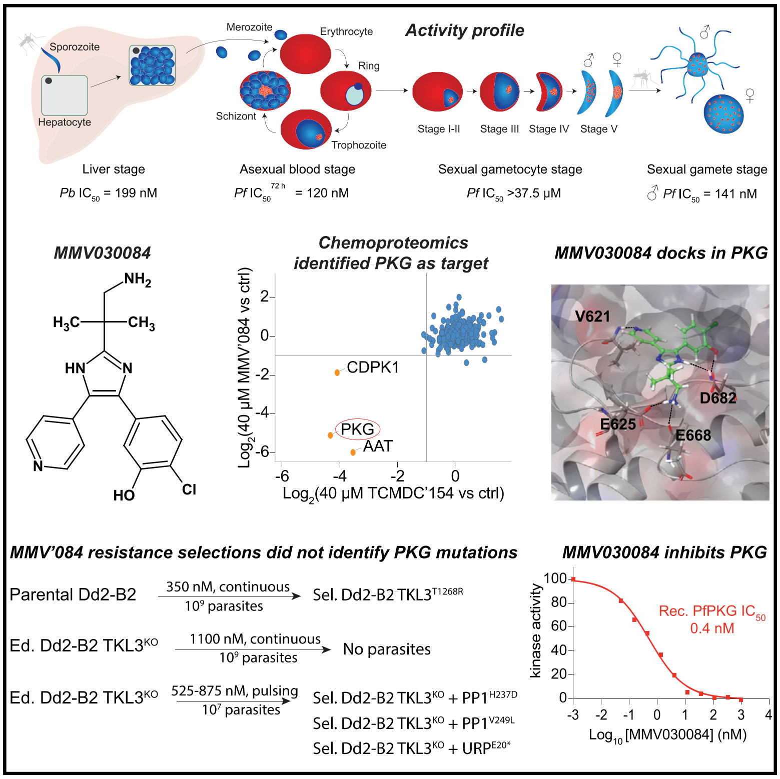

Highlights

- MMV030084 inhibits *P. falciparum* liver and asexual blood stages and male gametes
- Proteomic and conditional knockdown studies identified PfPKG as the target
- Resistance selection studies identified TKL3 as a low-level resistance mediator
- PKG is a promising resistance-refractory target for antimalarial drug development

Authors

Manu Vanaerschot, James M. Murithi, Charisse Flerida A. Pasaje, ..., Marcus C.S. Lee, Jacquin C. Niles, David A. Fidock

Correspondence

df2260@cumc.columbia.edu

In Brief

Vanaerschot et al. report an antimalarial, MMV030084, with potent antiplasmodial activity against all stages of human infection by *Plasmodium falciparum*. Metabolomic, phosphoproteomic, chemoproteomic, and gene-editing studies identified cGMP-dependent protein kinase (PKG) as the primary target, which did not mutate under selective drug pressure.

Vanaerschot et al., 2020, Cell Chemical Biology *27*, 806–816
July 16, 2020 © 2020 The Author(s). Published by Elsevier Ltd.
https://doi.org/10.1016/j.chembiol.2020.04.001

CellPress
OPEN ACCESS

Cell Chemical Biology

Article

Inhibition of Resistance-Refractory *P. falciparum* Kinase PKG Delivers Prophylactic, Blood Stage, and Transmission-Blocking Antiplasmodial Activity

Manu Vanaerschot,[1] James M. Murithi,[1] Charisse Flerida A. Pasaje,[2] Sonja Ghidelli-Disse,[3] Louis Dwomoh,[4] Megan Bird,[5] Natasha Spottiswoode,[1] Nimisha Mittal,[6] Lauren B. Arendse,[7] Edward S. Owen,[8,9] Kathryn J. Wicht,[1] Giulia Siciliano,[10] Markus Bösche,[3] Tomas Yeo,[1] T.R. Santha Kumar,[1] Sachel Mok,[1] Emma F. Carpenter,[11] Marla J. Giddins,[12] Olalla Sanz,[13] Sabine Ottilie,[6] Pietro Alano,[10] Kelly Chibale,[7] Manuel Llinás,[8,9,14] Anne-Catrin Uhlemann,[12] Michael Delves,[15] Andrew B. Tobin,[4] Christian Doerig,[5,16] Elizabeth A. Winzeler,[6] Marcus C.S. Lee,[11] Jacquin C. Niles,[2] and David A. Fidock[1,12,17,*]

[1]Department of Microbiology and Immunology, Columbia University Irving Medical Center, New York, NY 10032, USA
[2]Department of Biological Engineering, Massachusetts Institute of Technology, Cambridge, MA 02139, USA
[3]Cellzome GmbH, GlaxoSmithKline, 69117 Heidelberg, Germany
[4]Centre for Translational Pharmacology, Institute of Molecular Cell and Systems Biology, University of Glasgow, Glasgow G12 8QQ, UK, Scotland
[5]Department of Microbiology, Monash University, Melbourne, VIC 3800, Australia
[6]School of Medicine, University of California San Diego, La Jolla, CA 92093, USA
[7]Drug Discovery and Development Centre (H3D), South African Medical Research Council Drug Discovery and Development Research Unit, Department of Chemistry & Institute of Infectious Disease and Molecular Medicine, University of Cape Town, Rondebosch 7701, South Africa
[8]Department of Biochemistry and Molecular Biology, Pennsylvania State University, University Park, PA 16801, USA
[9]Huck Center for Malaria Research, Pennsylvania State University, University Park, PA 16802, USA
[10]Dipartimento di Malattie Infettive, Istituto Superiore di Sanità, Rome, Italy
[11]Wellcome Sanger Institute, Wellcome Genome Campus, Hinxton, Cambridgeshire, UK
[12]Division of Infectious Diseases, Columbia University Irving Medical Center, New York, NY 10032, USA
[13]Diseases of the Developing World Global Health Pharma Unit, GlaxoSmithKline, 28760 Tres Cantos, Spain
[14]Department of Chemistry, Pennsylvania State University, University Park, PA 16802, USA
[15]Department of Infection Biology, London School of Hygiene and Tropical Medicine, London WC1E 7HT, UK
[16]School of Health and Biomedical Sciences, RMIT University, Bundoora VIC 3083, Australia
[17]Lead Contact
*Correspondence: df2260@cumc.columbia.edu
https://doi.org/10.1016/j.chembiol.2020.04.001

SUMMARY

The search for antimalarial chemotypes with modes of action unrelated to existing drugs has intensified with the recent failure of first-line therapies across Southeast Asia. Here, we show that the trisubstituted imidazole MMV030084 potently inhibits hepatocyte invasion by *Plasmodium* sporozoites, merozoite egress from asexual blood stage schizonts, and male gamete exflagellation. Metabolomic, phosphoproteomic, and chemoproteomic studies, validated with conditional knockdown parasites, molecular docking, and recombinant kinase assays, identified cGMP-dependent protein kinase (PKG) as the primary target of MMV030084. PKG is known to play essential roles in *Plasmodium* invasion of and egress from host cells, matching MMV030084's activity profile. Resistance selections and gene editing identified tyrosine kinase-like protein 3 as a low-level resistance mediator for PKG inhibitors, while PKG itself never mutated under pressure. These studies highlight PKG as a resistance-refractory antimalarial target throughout the *Plasmodium* life cycle and promote MMV030084 as a promising *Plasmodium* PKG-targeting chemotype.

INTRODUCTION

Plasmodium falciparum (*Pf*) malaria, transmitted to humans through the bites of *Anopheles* mosquitoes, kills over 400,000 individuals each year, mostly young African children (WHO, 2019). Rapid diagnosis, effective treatment, and vector control have significantly reduced mortality and morbidity rates since 2000, but this progress has stalled in recent years. In addition, a major concern is the emergence and spread in Southeast Asia of *Pf* parasites resistant to artemisinins and partner drugs used in artemisinin-based combination therapies (Ross and Fidock, 2019; van der Pluijm et al., 2019). Consequently, there is an urgent need to complement the current arsenal of failing antimalarials with additional chemotypes having novel modes of action.

Human infection begins when *Plasmodium* sporozoites delivered during a mosquito bite migrate to the liver to undergo clonal replication in hepatocytes, generating thousands of merozoites able to infect red blood cells (RBCs) (Cowman et al., 2016). In RBCs, parasites develop over ~48 h from a ring into a trophozoite and eventually a schizont that contains 8-24 merozoites. Upon

 © 2020 The Author(s). Published by Elsevier Ltd.
This is an open access article under the CC BY license (http://creativecommons.org/licenses/by/4.0/).

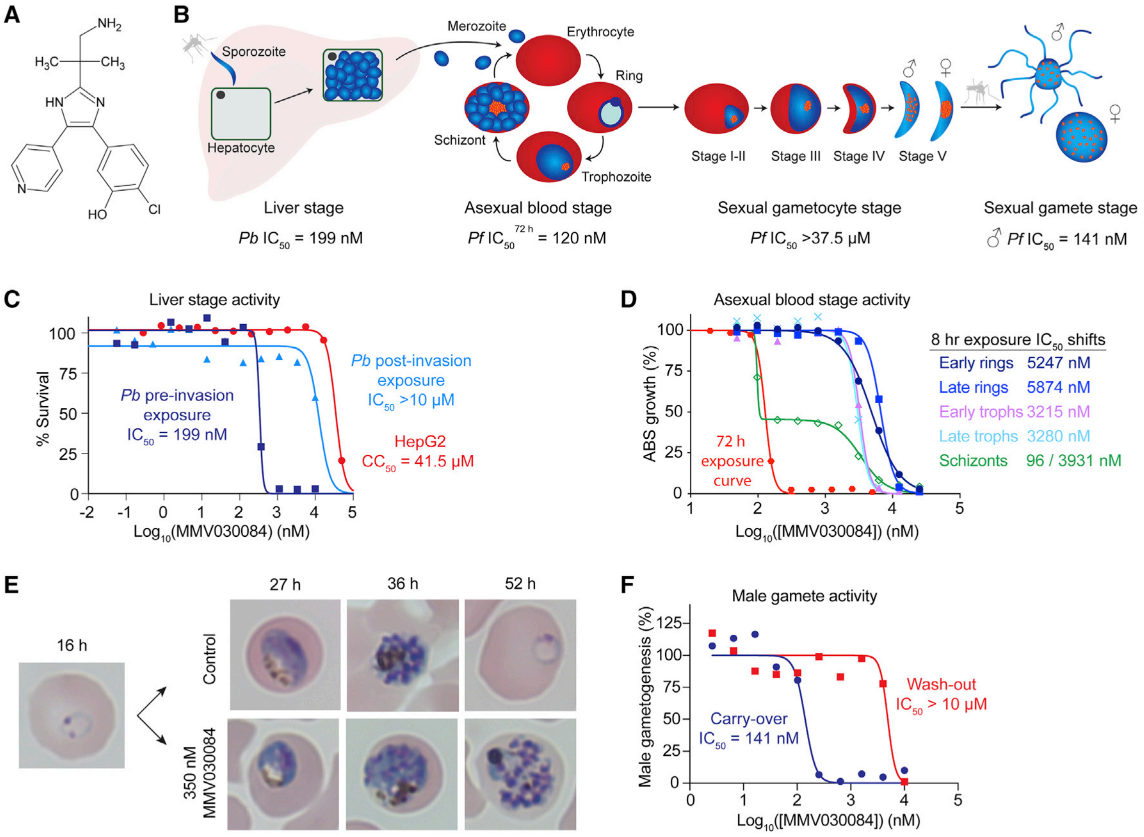

Figure 1. MMV030084 Has Prophylactic, Anti-ABS, and Transmission-Blocking Potential

(A) Structure of MMV030084, a trisubstituted imidazole.

(B) Overall activity profile of MMV030084.

(C) MMV030084 inhibited invasion of *P. berghei* ANKA parasites into HepG2 liver cells.

(D) Compound dose-response assays with ABS parasite stages, highly synchronized to within 3-h periods of development, indicated that MMV030084 is highly active only against synchronized schizonts. Minimal activity was observed against rings and trophozoites.

(E) Microscopy studies confirmed that MMV030084 is active against schizonts, showing the profile of an egress inhibitor.

(F) Male gamete formation is inhibited by MMV030084 in the presence of compound, but not when compound is washed out before gamete formation.

lysing their host RBCs, egressed merozoites invade other RBCs to perpetuate the asexual blood stage (ABS) cycle that causes all clinical manifestations of malaria. Some ABS parasites will develop into sexual gametocytes that can continue development in mosquitoes after ingestion during a blood meal. In the mosquito midgut, male and female gametocytes will develop into gametes, which fuse to form zygotes. These develop into ookinetes and later oocysts, which undergo meiosis and release thousands of sporozoites ready to infect a human host.

This complex life cycle provides opportunities to develop antimalarials with prophylactic (liver stage), curative (ABS), or transmission-blocking (gametocytes and/or gametes) activity. Screening platforms using *Pf* or the rodent-infecting *P. berghei* (*Pb*) species at different developmental stages have identified thousands of hits with potent activity (Antonova-Koch et al., 2018; Gamo et al., 2010; Plouffe et al., 2016; Van Voorhis et al., 2016). However, only rarely are the targets of these hits known, and even less frequently are compounds found that show prophylactic, anti-ABS, and transmission-blocking activity. In addition, it is critical to ensure that antimalarial therapies have a high barrier to resistance development by the parasite (Blasco et al., 2017; Phillips et al., 2017).

Here, we report the compound MMV030084, which shows potent activity against liver, asexual, and sexual blood stage development. Using chemoproteomic and genetic tools, we identified and validated cGMP-dependent protein kinase (PfPKG) as the target. *In vitro* resistance, mediated by tyrosine kinase-like protein 3 (TKL3), was low-level, making MMV030084 a promising candidate antimalarial for further development.

RESULTS

MMV030084 Displays Prophylactic, Anti-ABS, and Transmission-Blocking Antiplasmodial Activity

We first profiled the antiplasmodial life-cycle activity of MMV030084, a trisubstituted imidazole identified by the Medicines for Malaria Venture (MMV) (Figures 1A and 1B). In liver stage assays using luciferase-expressing *Pb* parasites and host HepG2 liver cells, MMV030084 showed a 50% inhibitory concentration (IC_{50}) of 199 nM (Figure 1C). This inhibitory activity peaked during parasite invasion, as indicated by experiments in which exposure started at different time points (Figure 1C). Against HepG2 liver cells, MMV030084 showed a 50% cytotoxic

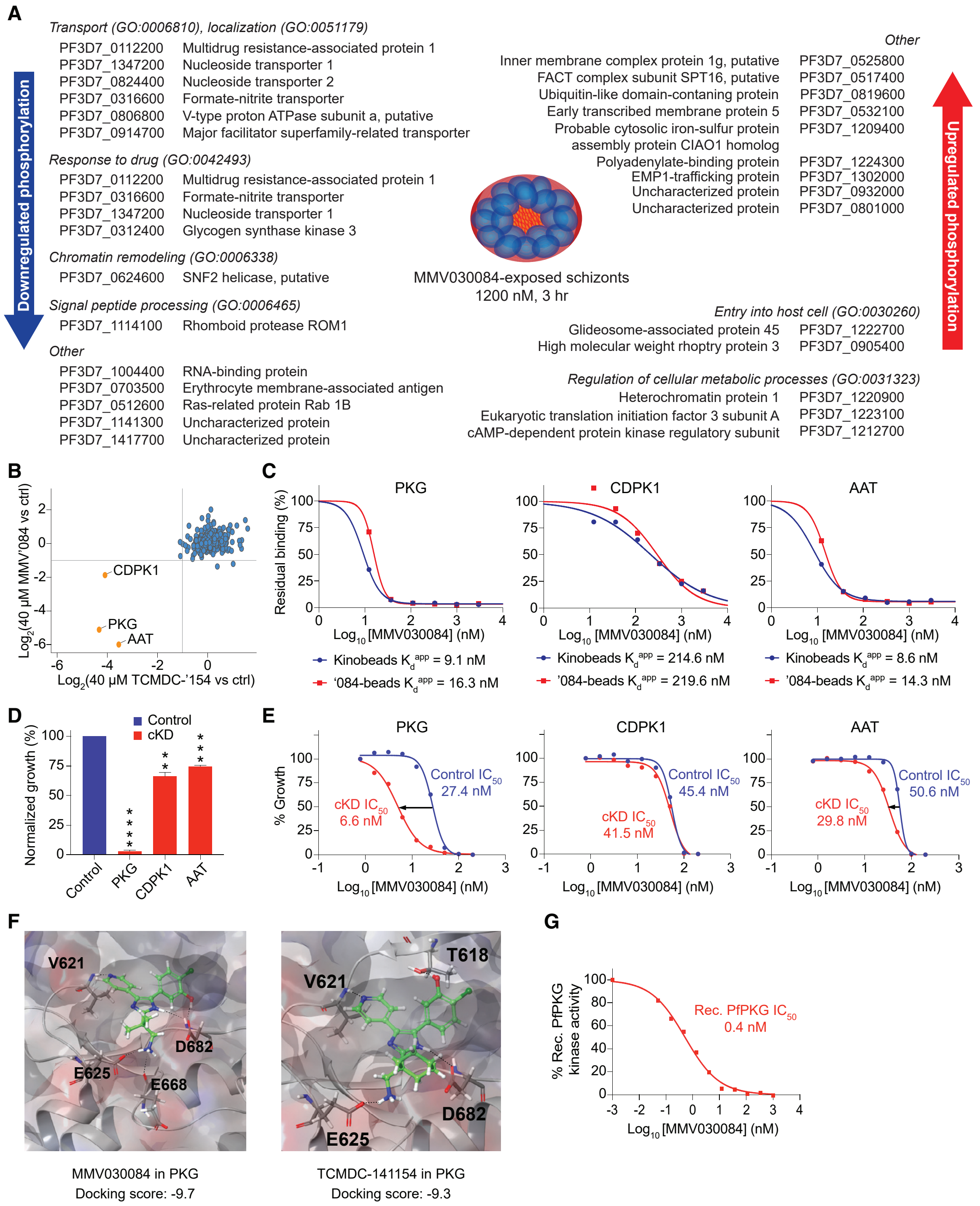

Figure 2. Identification and Validation of PKG as the Primary Target of MMV030084

(A) MMV030084-exposure of schizonts affected the phosphorylation status of several peptides in various pathways and provided evidence indicating that MMV030084 likely targets a kinase that is central to *Pf* parasite metabolism.

(B) PKG, CDPK1, and AAT emerged as candidate targets in competitive chemoproteomic studies using bead-bound compounds on treated and untreated (control) parasite lysates (Table S1 (E1-3)).

(C) Exposing MMV030084-treated parasite lysates to a panel of bead-immobilized kinase inhibitors (Kinobeads) or bead-immobilized MMV030084 ('084-beads) identified the lowest K_d^{app} values for PKG, CDPK1, and AAT. Results shown are the mean of two repeated experiments, except for AAT, which was detected in only one of the experiments (Table S1 (E1-3)).

(D) cKD of PKG resulted in the largest growth defect compared with control conditions, with cKDs of CDPK1 and AAT affecting parasite growth to a lesser extent. Error bars indicate the SEM based on two independently repeated experiments with technical duplicates. p values are based on unpaired t tests (Table S1 (F)).

(legend continued on next page)

concentration (CC_{50}) of 41.5 μM, illustrating low toxicity and high selectivity for antiplasmodial activity (Figure 1C).

Against *Pf* ABS parasites, MMV030084 showed an IC_{50} of 109 nM against the drug-sensitive 3D7-A10 line, and an IC_{50} of 120 nM against multidrug-resistant Dd2-B2 parasites in a 72-h growth inhibition assay (Figure 1D). We then explored the timing of MMV030084 inhibition of 3D7-A10 parasites during ABS development by exposing synchronized parasites at specific 8-h intervals. MMV030084 activity peaked against schizonts, with a biphasic survival curve that showed a first shift at 96 nM, a plateau phase at ~58% survival, and a second shift at 3,932 nM (Figure 1D; Table S1 (A)). This biphasic curve suggests the presence of primary and secondary targets, or some incomplete synchronicity in the schizont preparations. MMV030084-treated rings and trophozoites showed average IC_{50} values of 5,561 and 3,248 nM, respectively, with monophasic survival curves. These results were further validated by light microscopy, showing that ABS development was halted at the schizont stage when ring-stage parasites were exposed to 350 nM of MMV030084 (approximately 3× IC_{50}; Figure 1E).

We also tested a panel of MMV030084 analogs (Figure S1A) from the Tres Cantos Antimalarial Set (Gamo et al., 2010). MMV030734, included in the MMV Pathogen Box compound library, showed a 72-h ABS IC_{50} of 65 nM. Other analogs from this set showed IC_{50} values between 86 and 395 nM (Table S1 (B)). Among these analogs that include known kinase inhibitors, TCMDC-141334 was earlier reported to be a B-raf kinase inhibitor (Gamo et al., 2010).

MMV030084 proved inefficacious against both early and late stage NF54 gametocytes, showing IC_{50} values >40 μM in CBG99-luciferase assays (Siciliano et al., 2017) and saponin lysis-based sexual stage assays (Plouffe et al., 2008). Nonetheless, MMV030084 inhibited male gamete exflagellation at an IC_{50} of 141 nM when gametocytes were stimulated to develop into male gametes that egress from their host RBCs in the presence of the compound (Figure 1F). This activity was lost when MMV030084 was washed out before gamete stimulation, consistent with the lack of gametocyte killing. Female gamete formation assay data provided ambiguous results and could not clearly identify activity of MMV030084 against female gametes (Figure S2).

Exploratory Metabolomics and Phosphoproteomics Identify a Novel Mode of Action and Implicates the Kinase-Inhibitory Activity of MMV030084

We first assessed the impact of MMV030084 on the parasite's metabolic pathways using an exploratory metabolomics study on trophozoites and the more MMV030084-susceptibile schizonts. MMV030084 induced only minor changes within the set of detected metabolites in both stages (Table S1 (C)). This metabolic profile was distinct from inhibitors known to affect targets such as hemoglobin catabolism, the mitochondrial electron transport chain, pyrimidine biosynthesis, or cellular homeostasis (Murithi et al., 2019).

Since the structure of MMV030084 is reminiscent of kinase inhibitors, we also analyzed the impact of MMV030084 exposure (1,200 nM, equivalent to 10× IC_{50}, for 3 h) on the phosphoproteome of Dd2-B2 *Pf* schizonts. Results showed that MMV030084 exposure reduced the phosphorylation status of 16 proteins by more than 35% compared with untreated control conditions (Figure 2A; Table S1 (D1,2)). These proteins included nucleoside transporters 1 and 2, Ras-related protein Rab-1B, multidrug resistance-associated protein 1, vacuolar proton-translocating ATPase subunit a, erythrocyte membrane-associated antigen, the *Pf* formate-nitrite transporter, rhomboid-like proteins, SNF2 helicase, glycogen synthase kinase 3, an RNA-binding protein, and three uncharacterized proteins including a transporter. Conversely, 15 proteins showed increased phosphorylation levels after MMV030084 treatment compared with untreated conditions. These included a ubiquitin-like domain-containing protein, glideosome-associated protein 45, EMP1-trafficking protein, early transcribed membrane protein 5, high molecular weight rhoptry protein 3, heterochromatin protein 1, eukaryotic translation initiation factor 3 subunit A, inner membrane complex protein 1g, probable cytosolic iron-sulfur protein assembly protein CIAO1 homolog, protein dopey homolog, FACT complex subunit SPT16, cAMP-dependent protein kinase regulatory subunit, polyadenylate-binding protein, and two uncharacterized proteins (Figure 2A; Table S1 (D1,2)). The multitude of proteins and pathways that are affected suggested that MMV030084 targets one or more kinases central to the parasite's cellular and developmental regulation.

Chemoproteomics Identify PKG, CDPK1, and AAT as Putative Targets of MMV030084

We subsequently performed competitive chemoproteomics to identify binding targets of MMV030084. Our initial approach involved MMV030084 and its analog TCMDC-141154. The latter was chosen based on its availability, structural relatedness, and the presence of a primary amine moiety, which made it suitable for chemoproteomic profiling as we could attach this compound to a bead matrix without further modification. Both compounds were attached to an NHS-activated Sepharose matrix via their primary amine moiety and then incubated with parasite lysates that had been exposed to either 40 μM of the same compound or a vehicle control. cGMP-dependent protein kinase (PKG) (PF3D7_1436600; UniProtKB: Q8I719), a putative amino acid transporter (AAT) (PF3D7_1231400; UniProtKB: Q8I5A6), and calcium-dependent protein kinase 1 (CDPK1) (PF3D7_0217500; UniProtKB: P62344) were identified as the sole three proteins that were pulled down in both the MMV030084 and TCMDC-141154 competitive chemoproteomic experiments (Figure 2B; Table S1 (E1-3)).

(E) cKD of PKG increased parasite susceptibility to MMV030084, while cKD of CDPK1 or AAT caused no or minor differences in MMV030084 susceptibility versus control conditions. Results shown are based on four independently repeated experiments with technical duplicates (Table S1 (G)).

(F) MMV030084 and its analogs dock well into the crystal structure of PKG (Table S1 (H)). H-bonding residues are highlighted in the docking graphs.

(G) MMV030084 potently inhibited the kinase activity of recombinant PfPKG *in vitro*. IC_{50} data are based on three independently repeated experiments with technical duplicates. MMV'084, MMV030084; TCMDC'154, TCMDC-141154; ctrl, control; PKG, cGMP-dependent protein kinase (PF3D7_1436600); CDPK1, calcium-dependent protein kinase 1 (PF3D7_0217500); AAT, putative amino acid transporter (PF3D7_1231400); K_d^{app}, apparent dissociation constant; cKD, conditional knockdown; SEM, standard error of the mean. *$p < 0.05$, **$p < 0.01$, ***$p < 0.001$, ****$p < 0.0001$.

Our second approach involved parasite lysates being exposed to different concentrations of MMV030084 and subsequently incubated with a panel of bead-immobilized, known kinase inhibitors (Kinobeads assay) or with bead-immobilized MMV030084. Five independent experiments showed that PKG and CDPK1 were consistently subject to the highest level of competition between the beads and MMV030084, with apparent dissociation constants (K_d^{app}) below the lowest tested concentration of 0.1 μM (Table S1 (E1-3)). Two follow-up experiments using lower MMV030084 concentrations yielded more accurate K_d^{app} values of 9–16 nM for PKG and 215–220 nM for CDPK1, highlighting PKG as the primary candidate target of MMV030084 (Figure 2C; Table S1 (E1-3)). This assay also identified AAT as a putative binding partner of MMV030084 in one of the two follow-up experiments, at levels similar to PKG. We note that AAT was identified from only 2 to 3 peptides from the same region in the 229-kDa protein, whereas PKG was identified by over 30 peptides, suggesting that AAT may be a binding partner of PKG or CDPK1 but not of MMV030084 itself.

The same chemoproteomics approach was also applied to human mixed tissue extracts to test possible cross-reactivity of MMV030084 with human proteins. Of the 280 human kinases that were identified in the assay, seven showed K_d^{app} values < 100 nM (10× the K_d^{app} for PKG): receptor interacting serine/threonine kinase 2 (K_d^{app} = 4.5 nM), protein kinase N3 (K_d^{app} = 8.5 nM), nemo-like kinase (K_d^{app} = 20 nM), transforming growth factor β receptor 1 (K_d^{app} = 20 nM), testis-associated actin remodeling kinase 2 (K_d^{app} = 25 nM), transforming growth factor β receptor 2 (K_d^{app} = 35 nM), and casein kinase 1δ (K_d^{app} = 95 nM) (Table S1 (E1-3)).

Conditional Knockdowns, Molecular Modeling, and Kinase Inhibition Assays Validate PKG as a Primary Target for MMV030084's Antiplasmodial Activity

To further explore PKG, CDPK1, and AAT as candidate targets of MMV030084, we generated conditional knockdown (cKD) lines for each of these genes. Using CRISPR/Cas9, Tet repressor protein aptamers were introduced at the 3′ ends of these genes so that translation could be regulated with anhydrotetracycline (aTc) (Ganesan et al., 2016; Nasamu et al., 2019). High aTc levels allow translation, while low aTc levels result in a knockdown of the gene of interest. cKDs of PKG and CDPK1 were functionally validated by western blot (Figure S3). We confirmed the presence of the cKD machinery in the AAT cKD line by Sanger sequencing, as AAT protein levels were too low in standard conditions to validate by western blot.

We first assessed the essentiality of PKG, CDPK1, and AAT for *Pf* ABS by assessing parasite growth in the presence (50 nM) or absence of aTc. This showed that 97%, 34%, and 25% of parasite growth was inhibited under PKG, CDPK1, and AAT knockdown conditions, respectively, compared with controls (Figure 2D; Table S1 (F)). We then evaluated parasite susceptibility to MMV030084, and MMV030734 under knockdown versus wild-type conditions. Dose-response growth inhibition data revealed that parasites were sensitized 4.2-fold to MMV030084 when PKG was knocked down (Figure 2E; Table S1 (G)), consistent with the expected sensitization to a compound when its target is knocked down (Istvan et al., 2019). The rationale being that less compound is required to achieve growth inhibition by binding its target if already there is less functional target in the parasite. A similar trend, albeit to a lesser degree (1.7-fold decreased IC_{50}), was observed under AAT cKD conditions. No change in MMV030084 susceptibility was observed when CDPK1 was knocked down (Figure 2E). Similar results were obtained for the Pathogen Box analog MMV030734 (Table S1 (G)).

Given that PKG emerged as the primary candidate target for MMV030084 based on cKD data and that its crystal structure is available (PDB: 5DYK [Baker et al., 2017]), we modeled MMV030084 and several of its analogs into the ATP-binding pocket of PKG. This showed strong docking of MMV030084 and its analogs MMV030734 and TCMDC-141154 in PKG with scores less than −9.3 and the formation of stable H-bonds with residues V621, E625, and D682 for all tested compounds as confirmed by molecular dynamics studies (Figure 2F). Other MMV030084 analogs also docked well into the crystal structure of PKG (Table S1 (H)). Kinase inhibition assays using recombinant PfPKG indicated a mean IC_{50} of 0.4 nM for MMV030084, indicating potent *in vitro* inhibition of PKG activity by MMV030084 (Figure 2G).

To further confirm that CDPK1 is not the primary target of MMV030084, we tested a previously generated CDPK1 T145M gatekeeper mutant line, generated in NF54 parasites (Bansal et al., 2018), for its susceptibility to MMV030084. This gatekeeper residue mutation reduces accessibility to the small hydrophobic pocket adjoining the ATP-binding site that MMV030084 is predicted to extend into and should, therefore, reduce sensitivity of CDPK1 to the inhibitor. There was no change in IC_{50} to MMV030084 in the CDPK1^{T145M} line compared with its parental control (mean IC_{50} values of 112 nM for the wild-type NF54 parental line versus 90 nM for the CDPK1^{T145M} mutant line).

Selection Studies Reveal Lack of Mutation in the PKG Target and Identify Low-Grade Resistance Mediated by TKL3

To identify possible resistance mechanisms of MMV030084, we performed several rounds of *in vitro* MMV030084 resistance selections (Figure 3A). Continuous exposure of 1 × 10^9 Dd2-B2 parasites, in triplicate, to 350 nM (3× IC_{50}) of MMV030084 yielded resistant parasites that showed a 2.9-fold IC_{50} shift compared with the parental line (Figures 3A and 3B; Table S1 (I)). Whole-genome sequencing of resistant clones obtained by limited dilution identified a shared T1268R mutation located outside of the kinase catalytic domain of tyrosine kinase-like protein 3 or TKL3 (PF3D7_1349300) in all MMV030084-resistant lines (Figure 3B). To validate this finding, T1268R was introduced into wild-type Dd2-B2 parasites by CRISPR/Cas9 genetic editing, yielding the edited (ed.) Dd2-B2 TKL3^{T1268R} line. This line showed a 2.6-fold IC_{50} shift to MMV030084, similar to the MMV030084-selected line and validating TKL3 as a MMV030084-resistance mediator (Figure 3B; Table S1 (I)). The introduction of the I1250M mutation, a natural variant present in 3D7, into wild-type Dd2-B2 conferred a similar 2.5-fold IC_{50} shift, but provided no additional resistance when introduced in the edited Dd2-B2 TKL3^{T1268R} line. Using the same editing strategy, we also introduced a stop codon and a frameshift at the T1268 position to produce a truncated TKL3 knockout lacking the last 539 C-terminal amino acids (termed ed. Dd2-B2

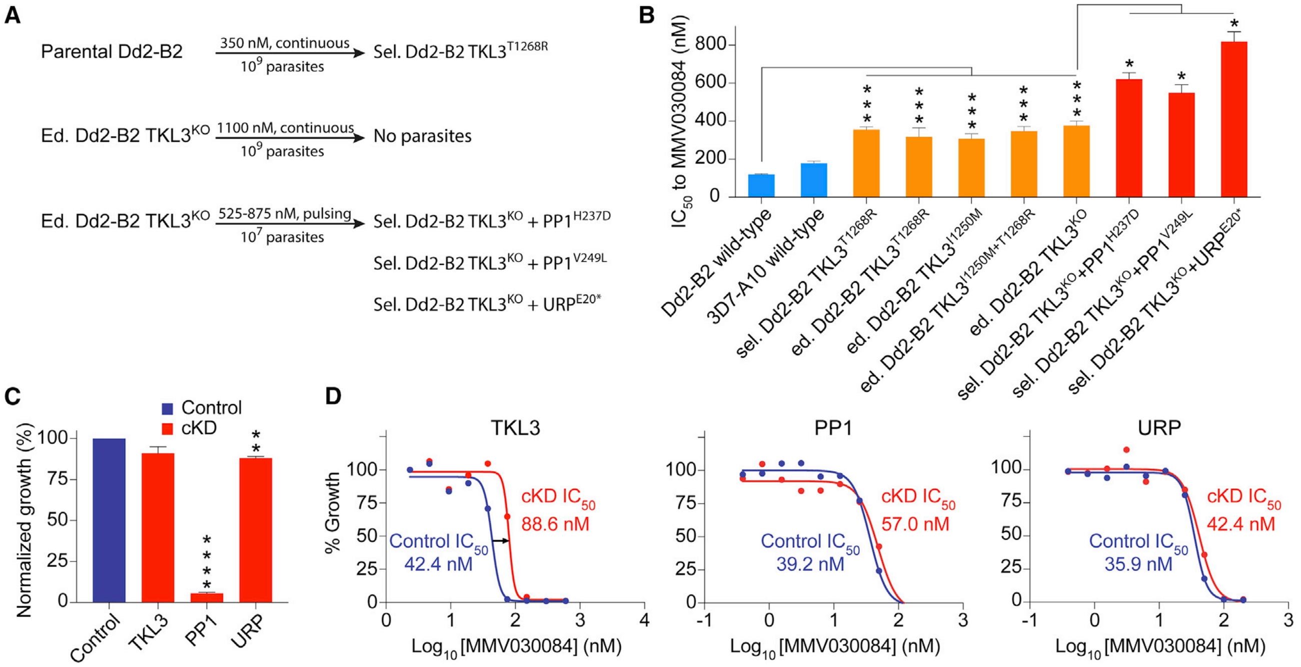

Figure 3. Resistance Selections with Cultured *Pf* ABS Parasites Identify TKL3, PP1, and URP as Low-Level Resistance Mediators for MMV030084

(A) Outline of the MMV030084 resistance selection attempts that identified TKL3, PP1, and URP as candidate resistance mediators.

(B) Susceptibility of the selected and confirmatory gene-edited clones to MMV030084. Error bars indicate the SEM based on ≥ 4 independently repeated experiments with technical duplicates. p values are based on Mann-Whitney U tests (Table S1 (I)).

(C) Parasite growth is only minimally inhibited by cKD of TKL3 and URP, while cKD of PP1 renders ABS parasites effectively non-viable (Table S1 (F)). Error bars indicate the SEM based on two independently repeated experiments with technical duplicates. p values are based on unpaired t tests.

(D) cKD of TKL3 decreased parasite susceptibility to MMV030084, whereas cKD of PP1 and URP showed no effect. Error bars indicate the SEM based on two independently repeated experiments with technical duplicates. p values are based on unpaired t tests (Table S1 (G)). Sel., selected; Ed., CRISPR/Cas9 edited; aTc, anhydrotetracycline; TKL3, tyrosine kinase-like protein 1 (PF3D7_1349300); PP1, protein phosphatase 1 (PF3D7_1414400); URP, ubiquitin regulatory protein (PF3D7_0808300); cKD, conditional knockdown; *$p < 0.05$, **$p < 0.01$, ***$p < 0.001$, ****$p < 0.0001$.

$TKL3^{KO}$). This knockout showed a 3.1-fold IC_{50} shift to MMV030084, similar to the other edited lines (Figure 3B; Table S1 (I)).

A TKL3 cKD line, engineered using the same strategy as mentioned earlier for PKG, showed only a 9% reduction in parasite growth in the absence of aTc (Figure 3C; Table S1 (F)). In addition, the TKL3 knockdown induced a 2.1-fold increase in IC_{50} to MMV030084 compared with its isogenic control (Figure 3D; Table S1 (G)). This finding is opposite to the IC_{50} decrease usually observed for drug targets.

To further assess whether MMV030084 can directly inhibit TKL3 activity, we used a radioactive *in vitro* kinase assay with a recombinant TKL3 construct containing the kinase domain region. A concentration of 20 μM MMV030084 or other analogs yielded no reduction in TKL3 kinase activity (Figure S1B). Similar results were observed for compounds MMV030734, TCMDC-140762, and TCMDC-141079, indicating that MMV030084 and other compound analogs did not directly inhibit TKL3 kinase activity.

We performed additional selections with the edited Dd2-B2 $TKL3^{KO}$ parasites to obtain higher resistance levels and explore its genetic basis. Continuous exposure of 1×10^9 Dd2-B2 $TKL3^{KO}$ parasites to 1,100 nM of MMV030084 did not result in parasite recrudescence. A pulsing procedure in which parasites were exposed to gradually increasing levels of MMV030084 (525–875 nM), however, did result in parasites with an additional 1.5- to 2.2-fold IC_{50} shift compared with the parental edited Dd2-B2 $TKL3^{KO}$ line (Figure 3B; Table S1 (I)). These parasites were cloned and subjected to whole-genome sequencing. This analysis revealed mutations in either protein phosphatase 1 (PP1) (PF3D7_1414400) or a putative ubiquitin regulatory protein (URP) (PF3D7_0808300). H237D and V249L in PP1 were observed in parasites that displayed an average 1.6-fold shift, whereas the E20* mutation in URP was associated with a 2.2-fold IC_{50} shift to MMV030084 compared with the parental ed. Dd2-B2 $TKL3^{KO}$ line (Figure 3B; Table S1 (I)).

cKDs of PP1 and URP in TKL3 wild-type parasites resulted in 95% and 12% mean reductions in parasite growth, respectively (Figures 3C and S3; Table S1 (F)). Neither the PP1 nor the URP cKD line showed a differential MMV030084 susceptibility compared with the isogenic controls (Figure 3D; Table S1 (G)), arguing against these proteins being direct targets of this compound.

DISCUSSION

Herein, we report the mode of action of and genetic basis of resistance to MMV030084, a trisubstituted imidazole with potent

activity against *Pf* ABS parasites (Gamo et al., 2010). We further characterized the activity profile of this compound, which primarily affects schizonts during ABS development, inhibiting parasite egress from infected RBCs (Figures 1D and 1E). Liver stage assays revealed potent inhibition of sporozoite host cell invasion without affecting liver trophozoite development (Figure 1C). MMV030084 also inhibited male gametogenesis *in vitro*, but did not kill mature gametocytes directly (Figure 1F). These results highlight MMV030084 as a valuable antimalarial candidate with prophylactic, anti-ABS, and transmission-blocking properties that fulfill many criteria of the target candidate and target product profiles for new antimalarials as set forth by MMV (Burrows et al., 2017).

MMV030084 contains a kinase inhibitor-like hinge binding motif (Urich et al., 2013). We therefore performed an exploratory phosphoproteomics analysis that identified a variety of proteins with a differential phosphorylation status in MMV030084-treated Dd2-B2 parasites versus DMSO-treated control conditions, without pinpointing a specific pathway (Figure 2A; Table S1 (D1,2)). Comprehensive target identification and validation studies, including chemoproteomics, translational cKDs, and modeling experiments highlighted PKG as the primary target of MMV030084. PKG showed the lowest K_d^{app} values in the Kinobeads assay (Figure 2C), and the largest growth defect and sensitization to MMV030084 in cKD conditions (Figures 2D and 2E). In addition, MMV030084 docked well into PKG's ATP-binding pocket (Figure 2F) and potently inhibited recombinant PKG activity *in vitro* (Figure 2G).

We identified PKG consensus motifs in peptides that were differentially phosphorylated under MMV030084 pressure, and also in peptides that showed no differences in phosphorylation status. These studies were performed using the established PfPKG consensus motif K/RR/KxpS/pT as well as the PfPKA consensus motif K/RxxpS/pT, based on previous reports of the extensive overlap in the primary amino acid sequences phosphorylated by PKG and PKA (Glass and Krebs, 1979; Francis et al., 2010). Detailed results revealing phosphorylation motifs and sites and consensus PKG sites are shown in Table S1 (D1,2).

Chemoproteomic experiments identified CDPK1 and AAT as putative MMV030084 targets (Figure 2B), yet these displayed either higher K_d^{app} values to MMV030084 (Figure 2C) or the knockdown experiments indicated only a partial growth defect and a minimal to no IC_{50} shift to MMV030084 in cKD versus control conditions for these proteins (Figure 2D). A gatekeeper mutant (T145M) of CDPK1 showed a similar MMV030084 susceptibility as its wild-type control, further indicating that CDPK1 is not the primary target responsible for this compound's whole-cell activity. Of interest, CDPK1 is a known cellular target of PKG (Alam et al., 2015) and the same is thought to be true for AAT. This may have caused these proteins to bind PKG and therefore be initially identified as putative targets through chemoproteomics. CDPK1 could potentially also act as a secondary target of MMV030084. A major role for CDPK1, however, is not supported by our cKD data that showed sensitization to MMV030084 upon reduced expression of PKG, but not of CDPK1 or AAT, coupled with our finding that the IC_{50} for MMV030084 was equivalent against a $CDPK1^{T145M}$ gatekeeper mutant line (Bansal et al., 2018) compared with its parental control. A similar small gatekeeper mutant, $CDPK1^{T145G}$, was used in an earlier study of imidazopyridazine compounds showing that the Class I category of inhibitors, which were highly potent against the CDPK1 enzyme, inhibited parasite growth via inhibition of PKG (Green et al., 2015).

In ABS parasites, PKG is known to play an essential role in regulating egress and invasion (Alam et al., 2015; Taylor et al., 2010). This kinase regulates the discharge of subtilisin-like serine protease (SUB1) from secretory organelles, called exonemes, into the parasitophorous vacuole (Collins et al., 2013). This discharge allows SUB1 to cleave and thereby activate enzymes involved in parasite egress and invasion. Studies with *Pf* and *P. berghei* show a key role for PKG in helping regulate multiple cellular Ca^{2+} signals essential for parasite development and transmission (Brochet et al., 2014). This extends to PKG regulation of Ca^{2+}-dependent protein kinase 5, an important egress mediator for ABS parasites (Dvorin et al., 2010). These crucial roles of PKG for parasite egress and invasion are in line with the schizont-specific activity observed for MMV030084 (Figures 1D and 1E).

PKG also plays an essential role in gametogenesis, regulating male gamete exflagellation and the rounding up of female gametocytes (McRobert et al., 2008). MMV030084 and some of its analogs have previously been identified as inhibitors of female gamete formation (Miguel-Blanco et al., 2017). In our assays, MMV030084 potently inhibited male exflagellation (Figure 1F), but showed ambiguous results regarding female gametocyte rounding up (Figure S2). At later stages of *Pf* development in the mosquito, PKG was also reported to control ookinete motility (Moon et al., 2009), allowing the parasite to cross the midgut of the mosquito and establish infection away from digestive enzymes in the bloodmeal.

The potent inhibition of hepatocyte invasion by MMV030084 (Figure 1C) is also in agreement with PKG's role in sporozoite motility and protein secretion required for invasion (Govindasamy et al., 2016). In addition, PKG was also found to regulate parasite egress from hepatocytes (Falae et al., 2010; Govindasamy et al., 2016). However, we were unable to assess this phenotype for MMV030084 since our assays studied liver stage development from invasion up until only late liver trophozoites.

Our review of the PKG literature identified structural similarity between MMV030084 and a trisubstituted pyrrole identified by Merck (named Compound 1), originally developed for *Eimeria* (Gurnett et al., 2002) and later reported to be active against *Toxoplasma gondii* and *P. berghei* (Diaz et al., 2006). We also observed a similar set of differentially phosphorylated proteins in our phosphoproteomic experiments as had been identified using an imidazopyridine from Merck (named Compound 2) that also inhibits PKG (Alam et al., 2015). Several of these proteins are involved in invasion/egress, including the schizont egress antigen-1, Myosin A, glideosome-associated protein 45, and the membrane skeletal protein IMC-1 (see Table S1 (D1)). We also observed common proteins identified as contributing to transport (nucleotide transporters 1 and 2), transcriptional regulation (putative RNA-binding protein), and chromatin regulation (putative SNF2 helicase). Compounds were also recently identified in a screen of the GSK Full Diversity Collection against recombinant PKG (Penzo et al., 2019). Of note, our chemoproteomics studies with MMV030084 ruled out cross-reactivity with other parasite kinases relevant for antiplasmodial activity (Figures 2B and 2C). These studies, as well as cKD assays (Figures 2D and

2E) and CDPK1 gatekeeper mutant studies, consistently pointed to PKG as the primary target of MMV030084.

To our knowledge, our study is the first to describe resistance mechanisms selected against a PfPKG inhibitor after drug selections with cultured *Pf* ABS parasites. Continuous exposure of Dd2-B2 parasites to 350 nM (or 3× IC_{50}) MMV030084 resulted in the T1268R mutation in TKL3, located on a phosphorylation site outside of its kinase domain. Using CRISPR/Cas9, we validated this mutation and the natural variant I1250M (present in 3D7 parasites) to confer an approximately 3-fold resistance shift to MMV030084 compared with parental wild-type Dd2-B2 parasites (Figure 3B). TKL3 is a kinase expressed in ABS parasites, gametocytes and sporozoites. In ABS parasites, it is predominantly expressed in schizonts and is detected throughout the cell but does not colocalize with known parasite organelles (Abdi et al., 2010). In merozoites, TKL3 localizes to elongated structures with bulbous ends. In immature gametocytes, this kinase co-localized to microtubules, suggesting a role in the subpellicular membrane complex that links cytoskeleton microtubules to the pellicular membrane. This is consistent with TKL3 possessing a MORN motif that is thought to anchor the cytoskeleton to membranous structures in *Toxoplasma gondii* (Abdi et al., 2010). Our results suggest that TKL3 is directly involved in pathways regulating parasite egress from RBCs, as shown by the ablation of the egress defect in ed. Dd2-B2 $TKL3^{T1268R}$ compared with wild-type Dd2-B2 parasites. However, TKL3 is not essential for the parasite as shown by the TKL3 cKD line showing only a minor growth defect versus isogenic controls (Figure 3C), and our successful efforts in producing a TKL3 knockout line (Figure 3B). Our data agree with recent genome-wide *P. berghei* reverse genetic screens (Gomes et al., 2015). These findings, however, contrast with earlier targeted single crossover (Abdi et al., 2010) and double homologous recombination experiments (Tewari et al., 2010), conducted in *Pf* and *P. berghei*, respectively, which had suggested essentiality or at least a major fitness defect of $TKL3^{KO}$ lines.

Interestingly, our ed. Dd2-B2 $TKL3^{KO}$ line showed a similar resistance level to MMV030084 as the ed. Dd2-B2 $TKL3^{T1268R}$ and $TKL3^{I1250M}$ lines (Figure 3B), suggesting that a 3-fold IC_{50} shift may be the maximum resistance level that TKL3 can mediate. The TKL3 cKD line exhibited decreased susceptibility to MMV030084 (Figure 3D), in contrast to the increased sensitivity that is usually observed in cKD lines for drug targets. In addition, kinase assays using recombinant truncated TKL3 showed no difference in phosphorylation levels upon exposure to MMV030084 or its analogs compared with control conditions (Figure 3E). A phosphoproteomics experiment comparing MMV030084-treated Dd2-B2 $TKL3^{KO}$ parasites with untreated Dd2-B2 $TKL3^{KO}$ parasites showed a similar profile as observed in Dd2-B2 wild-type parasites, with the exception that fewer peptides were observed to have increased phosphorylation levels in Dd2-B2 $TKL3^{KO}$ (Table S1 (D1,2)). These data provide evidence that TKL3 is solely a resistance mediator of MMV030084, and not its target. It is possible that TKL3 might activate MMV030084 to increase its potency, and that mutations or gene deletion leads to reduced drug potency and thus parasite resistance. A similar scenario was recently demonstrated with a *Pf* esterase and the antiplasmodial compound MMV011438 (Istvan et al., 2017). Alternatively, mutations or gene deletion in TKL3 might result in differential phosphorylation levels of one or more essential PKG substrates in the presence of MMV030084 that chemically inhibits PKG, thus enabling *Pf* resistance to this compound. The exact mechanism by which TKL3 contributes to MMV030084 resistance requires further experimentation.

We performed additional selections with the ed. Dd2-B2 $TKL3^{KO}$ parasites in order to obtain higher resistance levels and explore its genetic basis. Single-step selections failed to yield resistance parasites, but a lengthy pulsing protocol yielded parasites with a 1.5- to 2.2-fold IC_{50} shift compared with the parental ed. Dd2-B2 $TKL3^{KO}$ line. These parasites showed either H237D or V249L mutations in PP1 or the E20* (stop) mutation in URP. PP1 and URP cKD lines showed no major changes in susceptibility to MMV030084, indicating that both are resistance mediators but not direct targets of MMV030084.

PP1 is considered essential for the parasite's ABS development (Guttery et al., 2014), consistent with our observation of a large fitness defect under PP1 cKD conditions (Figure 3C). A recent study on the interactome of PP1 identified multiple interacting partners that are thought to determine PP1's final localization and specificity and make PP1 a central phosphatase regulating several diverse parasite pathways (Hollin et al., 2016). We could not identify a direct link between PP1 and PKG, TKL3, or URP. However, PP1 was found to be involved in egress by regulating the phosphorylation status of *Pf* skeleton binding protein 1 (PfSBP1) (Blisnick et al., 2006). PfSBP1 is a transmembrane protein located in the parasite's Maurer's clefts that regulates the stability of the host cell membrane, thereby facilitating egress of the parasite from the RBC, with hyperphosphorylation of PfSBP1 inhibiting egress. The H237D and V249L mutations could therefore result in a differential interaction of PP1 with its binding partners to overcome the inhibition of PKG by MMV030084 and its resulting egress defect.

The link between PKG and URP, however, is less straightforward. URP likely regulates ubiquitination and degradation of proteins by the proteasome, and it is reported to have phosphorylation sites at position S37 and S140 (Collins et al., 2014; Kumar et al., 2017). A recent report found that PKG phosphorylates RPT1, an AAA + ATPase present in the 19S regulatory unit of the proteasome, and regulates parasite proteolysis (Govindasamy et al., 2019). Interestingly, the same report highlighted the importance of proteolysis for invasion by sporozoites of hepatocytes. In line with these data, the E20* mutation in URP observed in our study might compensate for an invasion defect induced by PKG inhibition.

Importantly, no mutations in PKG conferring MMV030084 resistance were ever selected in any of our studies. We obtained only low-level resistance despite extensive efforts to elicit high-grade resistance. This likely reflects PKG's critical role in *Pf* ABS development. Altogether, our findings highlight PKG as a validated antimalarial target, and MMV030084 as a promising antimalarial chemotype active throughout the *Pf* life cycle.

SIGNIFICANCE

Knowledge of the target of candidate antimalarials is informative for drug development pipelines aiming to maximize potency and minimize toxicity. Here, we report the application of a multi-pronged target discovery pipeline to identify the molecular target and mode of action of MMV030084, a

molecule that we validated to have potential to prevent, treat, and limit malaria transmission. Results from chemoproteomic approaches, validated by gene-editing and conditional knockdown studies, revealed that cGMP-dependent protein kinase (PKG) is the primary antiplasmodial target. We also observed potent MMV030084-mediated inhibition of parasite egress from host red blood cells. This finding agrees with the essential role of PKG during cell-to-cell transitional stages of the *Plasmodium* parasite life cycle.

Because resistance development can be a major impediment for antimalarial efficacy, we also explored how *P. falciparum* parasites may become resistant to MMV030084. Resistance selections showed that the parasite could only develop low-level resistance, mediated by tyrosine kinase 3 (TKL3). TKL3 is not essential for the parasite, as shown with our knockout studies, but when mutated allows the parasite to achieve egress in the presence of MMV030084. Importantly, PKG itself never mutated during these long-term selections, highlighting that PKG inhibitors have a relatively low risk for high-grade resistance development, as compared with many other candidate antimalarials in development.

This study not only underscores the potential of PKG inhibitors for malaria control, but also provides an extended molecular toolbox to validate future analogs that may be developed.

STAR★METHODS

Detailed methods are provided in the online version of this paper and include the following:

- KEY RESOURCES TABLE
- RESOURCE AVAILABILITY
  - Lead Contact
  - Materials Availability
  - Data and Code Availability
- EXPERIMENTAL MODEL AND SUBJECT DETAILS
- METHOD DETAILS
  - Liver Stage Susceptibility Testing
  - Asexual Blood Stage Susceptibility Testing
  - Gametocyte Susceptibility
  - Dual Gamete Formation Assay
  - Metabolomics
  - Phosphoproteomics
  - Chemoproteomics
  - Generation of Constructs and Parasite Transfections for cKD Lines
  - Verification of Conditional Knockdown (cKD) Strategy
  - Growth Assays Using cKD Lines
  - Compound Susceptibility Assays Using cKD Lines
  - PfPKG Docking Studies
  - Recombinant PfPKG Inhibition Assay
  - Selections for MMV030084 Resistance
  - Whole-Genome Sequencing
  - CRISPR/Cas9 Editing of TKL3
  - Expression, Purification and Kinase Assay of WT and Kinase Dead Recombinant *Pf*TKL3 Kinase Domain
- QUANTIFICATION AND STATISTICAL ANALYSIS

SUPPLEMENTAL INFORMATION

Supplemental Information can be found online at https://doi.org/10.1016/j.chembiol.2020.04.001.

ACKNOWLEDGMENTS

This work was supported by the Bill and Melinda Gates Foundation that funds the Malarial Drug Accelerator (MalDA) consortium (OPP1054480 to E.A.W., D.A.F., M.C.S.L., and M.L., and OPP1162467 to J.C.N.), the Medicines for Malaria Venture (08/0015 to D.A.F.), the NIH (R01 AI090141 to E.A.W.) the Italian Ministries of Health and Foreign Affairs (ISARP2018-ISS to P.A.), and the LSHTM/Wellcome ISSF (to M.D.). K.C. gratefully acknowledges the support of the University of Cape Town, the South African Medical Research Council, the South African Research Chairs Initiative of the Department of Science and Innovation administered through the South African National Research Foundation, and the Global Challenges Research Fund (Synchrotron Techniques for African Research and Technology project). S.M. was supported by a Human Frontier Science Program long-term fellowship award. We sincerely thank the team at Cellzome for the chemoproteomic experiments, Drs. Louis Miller and Karthigayan Gunalan at the NIH for providing the CDPK1^{T145M} NF54 mutant line, and Dr. David Baker at the London School of Hygiene & Tropical Medicine, UK for providing the PfPKG expression construct. MMV030084 (TCMDC-140369) and MMV030734 (TCMDC-141019) were kindly provided by MMV, and the other TCMDC analogs (Figure S1A) were kindly provided by GlaxoSmithKline, Tres Cantos, Spain, through the MalDA consortium.

AUTHOR CONTRIBUTIONS

M.V. and J.M.M. performed ABS specificity assays and exposure studies. N.M., S.O., and E.A.W. performed liver stage assays. G.S., P.A., S.O., and E.A.W. assessed gametocytocidal activity of compounds. M.D. performed the dual gamete formation assay. E.S.O. and M.L. performed metabolomics experiments. L.D. and A.B.T. performed phosphoproteomic studies. S.G.-D. supervised the chemoproteomic studies that were performed at Cellzome with help from S.O. and B.M. C.F.A.P. and J.C.N. generated and phenotyped the cKD lines. K.J.W. performed PKG docking studies. L.B.A. and K.C. performed recombinant PfPKG kinase inhibition studies. M.V., J.M.M., and K.T.S. performed resistance selections. T.Y., S.M., M.J.G., and A.-C.U. performed whole-genome sequencing of these lines. M.C.S.L. and E.F.C. designed and produced CRISPR-Cas9 plasmids. M.V., J.M.M., N.S., and E.F.C. performed CRISPR-Cas9 editing experiments to validate resistance mediators. M.B. and C.D. performed recombinant TKL3 studies. M.V. compiled all the data and wrote the manuscript with J.M.M. and D.A.F., with input from other authors. All authors approved the final manuscript.

DECLARATION OF INTERESTS

The authors declare no competing interests.

Received: February 15, 2020
Revised: March 20, 2020
Accepted: March 31, 2020
Published: April 30, 2020

REFERENCES

Abdi, A., Eschenlauer, S., Reininger, L., and Doerig, C. (2010). SAM domain-dependent activity of PfTKL3, an essential tyrosine kinase-like kinase of the human malaria parasite *Plasmodium falciparum*. Cell Mol. Life Sci. *67*, 3355–3369.

Agrawal, S., Kumar, S., Sehgal, R., George, S., Gupta, R., Poddar, S., Jha, A., and Pathak, S. (2019). El-MAVEN: a fast, robust, and user-friendly mass spectrometry data processing engine for metabolomics. Methods Mol. Biol. *1978*, 301–321.

Alam, M.M., Solyakov, L., Bottrill, A.R., Flueck, C., Siddiqui, F.A., Singh, S., Mistry, S., Viskaduraki, M., Lee, K., Hopp, C.S., et al. (2015).

Phosphoproteomics reveals malaria parasite protein kinase G as a signalling hub regulating egress and invasion. Nat. Commun. *6*, 7285.

Allman, E.L., Painter, H.J., Samra, J., Carrasquilla, M., and Llinas, M. (2016). Metabolomic profiling of the malaria box reveals antimalarial target pathways. Antimicrob. Agents Chemother. *60*, 6635–6649.

Antonova-Koch, Y., Meister, S., Abraham, M., Luth, M.R., Ottilie, S., Lukens, A.K., Sakata-Kato, T., Vanaerschot, M., Owen, E., Jado, J.C., et al. (2018). Open-source discovery of chemical leads for next-generation chemoprotective antimalarials. Science *362*, aat9446.

Baker, D.A., Stewart, L.B., Large, J.M., Bowyer, P.W., Ansell, K.H., Jimenez-Diaz, M.B., El Bakkouri, M., Birchall, K., Dechering, K.J., Bouloc, N.S., et al. (2017). A potent series targeting the malarial cGMP-dependent protein kinase clears infection and blocks transmission. Nat. Commun. *8*, 430.

Bansal, A., Molina-Cruz, A., Brzostowski, J., Liu, P., Luo, Y., Gunalan, K., Li, Y., Ribeiro, J.M.C., and Miller, L.H. (2018). PfCDPK1 is critical for malaria parasite gametogenesis and mosquito infection. Proc. Natl. Acad. Sci. U S A *115*, 774–779.

Bantscheff, M., Eberhard, D., Abraham, Y., Bastuck, S., Boesche, M., Hobson, S., Mathieson, T., Perrin, J., Raida, M., Rau, C., et al. (2007). Quantitative chemical proteomics reveals mechanisms of action of clinical ABL kinase inhibitors. Nat. Biotechnol. *25*, 1035–1044.

Bantscheff, M., Hopf, C., Savitski, M.M., Dittmann, A., Grandi, P., Michon, A.M., Schlegl, J., Abraham, Y., Becher, I., Bergamini, G., et al. (2011). Chemoproteomics profiling of HDAC inhibitors reveals selective targeting of HDAC complexes. Nat. Biotechnol. *29*, 255–265.

Blasco, B., Leroy, D., and Fidock, D.A. (2017). Antimalarial drug resistance: linking *Plasmodium falciparum* parasite biology to the clinic. Nat. Med. *23*, 917–928.

Blisnick, T., Vincensini, L., Fall, G., and Braun-Breton, C. (2006). Protein phosphatase 1, a *Plasmodium falciparum* essential enzyme, is exported to the host cell and implicated in the release of infectious merozoites. Cell Microbiol. *8*, 591–601.

Brochet, M., Collins, M.O., Smith, T.K., Thompson, E., Sebastian, S., Volkmann, K., Schwach, F., Chappell, L., Gomes, A.R., Berriman, M., et al. (2014). Phosphoinositide metabolism links cGMP-dependent protein kinase G to essential Ca(2)(+) signals at key decision points in the life cycle of malaria parasites. PLoS Biol. *12*, e1001806.

Burrows, J.N., Duparc, S., Gutteridge, W.E., Hooft van Huijsduijnen, R., Kaszubska, W., Macintyre, F., Mazzuri, S., Mohrle, J.J., and Wells, T.N.C. (2017). New developments in anti-malarial target candidate and product profiles. Malar. J. *16*, 26.

Cevenini, L., Camarda, G., Michelini, E., Siciliano, G., Calabretta, M.M., Bona, R., Kumar, T.R., Cara, A., Branchini, B.R., Fidock, D.A., et al. (2014). Multicolor bioluminescence boosts malaria research: quantitative dual-color assay and single-cell imaging in *Plasmodium falciparum* parasites. Anal. Chem. *86*, 8814–8821.

Collins, C.R., Hackett, F., Strath, M., Penzo, M., Withers-Martinez, C., Baker, D.A., and Blackman, M.J. (2013). Malaria parasite cGMP-dependent protein kinase regulates blood stage merozoite secretory organelle discharge and egress. PLoS Pathog. *9*, e1003344.

Collins, M.O., Wright, J.C., Jones, M., Rayner, J.C., and Choudhary, J.S. (2014). Confident and sensitive phosphoproteomics using combinations of collision induced dissociation and electron transfer dissociation. J. Proteomics *103*, 1–14.

Cowman, A.F., Healer, J., Marapana, D., and Marsh, K. (2016). Malaria: biology and disease. Cell *167*, 610–624.

Deitsch, K., Driskill, C., and Wellems, T. (2001). Transformation of malaria parasites by the spontaneous uptake and expression of DNA from human erythrocytes. Nucleic Acids Res. *29*, 850–853.

Delves, M.J., Straschil, U., Ruecker, A., Miguel-Blanco, C., Marques, S., Dufour, A.C., Baum, J., and Sinden, R.E. (2016). Routine *in vitro* culture of *P. falciparum* gametocytes to evaluate novel transmission-blocking interventions. Nat. Protoc. *11*, 1668–1680.

Diaz, C.A., Allocco, J., Powles, M.A., Yeung, L., Donald, R.G., Anderson, J.W., and Liberator, P.A. (2006). Characterization of *Plasmodium falciparum* cGMP-dependent protein kinase (PfPKG): antiparasitic activity of a PKG inhibitor. Mol. Biochem. Parasitol. *146*, 78–88.

Dvorin, J.D., Martyn, D.C., Patel, S.D., Grimley, J.S., Collins, C.R., Hopp, C.S., Bright, A.T., Westenberger, S., Winzeler, E., Blackman, M.J., et al. (2010). A plant-like kinase in *Plasmodium falciparum* regulates parasite egress from erythrocytes. Science *328*, 910–912.

Falae, A., Combe, A., Amaladoss, A., Carvalho, T., Menard, R., and Bhanot, P. (2010). Role of *Plasmodium berghei* cGMP-dependent protein kinase in late liver stage development. J. Biol. Chem. *285*, 3282–3288.

Francis, S.H., Busch, J.L., Corbin, J.D., and Sibley, D. (2010). cGMP-dependent protein kinases and cGMP phosphodiesterases in nitric oxide and cGMP action. Pharmacol. Rev. *62*, 525–563.

Friesner, R.A., Banks, J.L., Murphy, R.B., Halgren, T.A., Klicic, J.J., Mainz, D.T., Repasky, M.P., Knoll, E.H., Shelley, M., Perry, J.K., et al. (2004). Glide: a new approach for rapid, accurate docking and scoring. 1. Method and assessment of docking accuracy. J. Med. Chem. *47*, 1739–1749.

Gamo, F.J., Sanz, L.M., Vidal, J., de Cozar, C., Alvarez, E., Lavandera, J.L., Vanderwall, D.E., Green, D.V., Kumar, V., Hasan, S., et al. (2010). Thousands of chemical starting points for antimalarial lead identification. Nature *465*, 305–310.

Ganesan, S.M., Falla, A., Goldfless, S.J., Nasamu, A.S., and Niles, J.C. (2016). Synthetic RNA-protein modules integrated with native translation mechanisms to control gene expression in malaria parasites. Nat. Commun. *7*, 10727.

Glass, D.B., and Krebs, E.G. (1979). Comparison of the substrate specificity of adenosine 3':5'-monophosphate- and guanosine 3':5'-monophosphate-dependent protein kinases. Kinetic studies using synthetic peptides corresponding to phosphorylation sites in histone H2B. J. Biol. Chem. *254*, 9728–9738.

Gomes, A.R., Bushell, E., Schwach, F., Girling, G., Anar, B., Quail, M.A., Herd, C., Pfander, C., Modrzynska, K., Rayner, J.C., et al. (2015). A genome-scale vector resource enables high-throughput reverse genetic screening in a malaria parasite. Cell Host Microbe *17*, 404–413.

Govindasamy, K., Jebiwott, S., Jaijyan, D.K., Davidow, A., Ojo, K.K., Van Voorhis, W.C., Brochet, M., Billker, O., and Bhanot, P. (2016). Invasion of hepatocytes by *Plasmodium* sporozoites requires cGMP-dependent protein kinase and calcium dependent protein kinase 4. Mol. Microbiol. *102*, 349–363.

Govindasamy, K., Khan, R., Snyder, M., Lou, H.J., Du, P., Kudyba, H.M., Muralidharan, V., Turk, B.E., and Bhanot, P. (2019). *Plasmodium falciparum* cyclic GMP-dependent protein kinase interacts with a subunit of the parasite proteasome. Infect. Immunol. *87*, 00518–00523.

Green, J.L., Moon, R.W., Whalley, D., Bowyer, P.W., Wallace, C., Rochani, A., Nageshan, R.K., Howell, S.A., Grainger, M., Jones, H.M., et al. (2015). Imidazopyridazine inhibitors of *Plasmodium falciparum* calcium-dependent protein kinase 1 also target cyclic GMP-dependent protein kinase and heat shock protein 90 to kill the parasite at different stages of intracellular development. Antimicrob. Agents Chemother. *60*, 1464–1475.

Gurnett, A.M., Liberator, P.A., Dulski, P.M., Salowe, S.P., Donald, R.G., Anderson, J.W., Wiltsie, J., Diaz, C.A., Harris, G., Chang, B., et al. (2002). Purification and molecular characterization of cGMP-dependent protein kinase from Apicomplexan parasites. A novel chemotherapeutic target. J. Biol. Chem. *277*, 15913–15922.

Guttery, D.S., Poulin, B., Ramaprasad, A., Wall, R.J., Ferguson, D.J., Brady, D., Patzewitz, E.M., Whipple, S., Straschil, U., Wright, M.H., et al. (2014). Genome-wide functional analysis of *Plasmodium* protein phosphatases reveals key regulators of parasite development and differentiation. Cell Host Microbe *16*, 128–140.

Hollin, T., De Witte, C., Lenne, A., Pierrot, C., and Khalife, J. (2016). Analysis of the interactome of the Ser/Thr protein phosphatase type 1 in *Plasmodium falciparum*. BMC Genomics *17*, 246.

Hughes, C.S., Foehr, S., Garfield, D.A., Furlong, E.E., Steinmetz, L.M., and Krijgsveld, J. (2014). Ultrasensitive proteome analysis using paramagnetic bead technology. Mol. Syst. Biol. *10*, 757.

Istvan, E.S., Das, S., Bhatnagar, S., Beck, J.R., Owen, E., Llinas, M., Ganesan, S.M., Niles, J.C., Winzeler, E., Vaidya, A.B., et al. (2019). *Plasmodium* Niemann-Pick type C1-related protein is a druggable target required for parasite membrane homeostasis. eLife *8*, 40529.

Istvan, E.S., Mallari, J.P., Corey, V.C., Dharia, N.V., Marshall, G.R., Winzeler, E.A., and Goldberg, D.E. (2017). Esterase mutation is a mechanism of resistance to antimalarial compounds. Nat. Commun. *8*, 14240.

Kumar, S., Kumar, M., Ekka, R., Dvorin, J.D., Paul, A.S., Madugundu, A.K., Gilberger, T., Gowda, H., Duraisingh, M.T., Keshava Prasad, T.S., et al. (2017). PfCDPK1 mediated signaling in erythrocytic stages of *Plasmodium falciparum*. Nat. Commun. *8*, 63.

McRobert, L., Taylor, C.J., Deng, W., Fivelman, Q.L., Cummings, R.M., Polley, S.D., Billker, O., and Baker, D.A. (2008). Gametogenesis in malaria parasites is mediated by the cGMP-dependent protein kinase. PLoS Biol. *6*, e139.

Miguel-Blanco, C., Molina, I., Bardera, A.I., Diaz, B., de Las Heras, L., Lozano, S., Gonzalez, C., Rodrigues, J., Delves, M.J., Ruecker, A., et al. (2017). Hundreds of dual-stage antimalarial molecules discovered by a functional gametocyte screen. Nat. Commun. *8*, 15160.

Moon, R.W., Taylor, C.J., Bex, C., Schepers, R., Goulding, D., Janse, C.J., Waters, A.P., Baker, D.A., and Billker, O. (2009). A cyclic GMP signalling module that regulates gliding motility in a malaria parasite. PLoS Pathog. *5*, e1000599.

Murithi, J.M., Owen, E.S., Istvan, E.S., Lee, M.C.S., Ottilie, S., Chibale, K., Goldberg, D.E., Winzeler, E.A., Llinas, M., Fidock, D.A., et al. (2019). Combining stage specificity and metabolomic profiling to advance antimalarial drug discovery. Cell Chem. Biol. *27*, 158–171.

Nasamu, A.S., Falla, A., Pasaje, C.F.A., Wall, B.A., Wagner, J.C., Ganesan, S.M., Goldfless, S.J., and Niles, J.C. (2019). An integrated platform for genome engineering and gene expression perturbation in *Plasmodium falciparum*. bioRxiv. https://doi.org/10.1101/816504.

Paquet, T., Le Manach, C., Cabrera, D.G., Younis, Y., Henrich, P.P., Abraham, T.S., Lee, M.C.S., Basak, R., Ghidelli-Disse, S., Lafuente-Monasterio, M.J., et al. (2017). Antimalarial efficacy of MMV390048, an inhibitor of *Plasmodium* phosphatidylinositol 4-kinase. Sci. Transl. Med. *9*, aad9735.

Penzo, M., de Las Heras-Duena, L., Mata-Cantero, L., Diaz-Hernandez, B., Vazquez-Muniz, M.J., Ghidelli-Disse, S., Drewes, G., Fernandez-Alvaro, E., and Baker, D.A. (2019). High-throughput screening of the *Plasmodium falciparum* cGMP-dependent protein kinase identified a thiazole scaffold which kills erythrocytic and sexual stage parasites. Sci. Rep. *9*, 7005.

Phillips, M.A., Burrows, J.N., Manyando, C., van Huijsduijnen, R.H., Van Voorhis, W.C., and Wells, T.N.C. (2017). Malaria. Nat. Rev. Dis. Primers *3*, 17050.

Plouffe, D., Brinker, A., McNamara, C., Henson, K., Kato, N., Kuhen, K., Nagle, A., Adrian, F., Matzen, J.T., Anderson, P., et al. (2008). *In silico* activity profiling reveals the mechanism of action of antimalarials discovered in a high-throughput screen. Proc. Natl. Acad. Sci. U S A *105*, 9059–9064.

Plouffe, D.M., Wree, M., Du, A.Y., Meister, S., Li, F., Patra, K., Lubar, A., Okitsu, S.L., Flannery, E.L., Kato, N., et al. (2016). High-throughput assay and discovery of small molecules that interrupt malaria transmission. Cell Host Microbe *19*, 114–126.

Ross, L.S., and Fidock, D.A. (2019). Elucidating mechanisms of drug-resistant *Plasmodium falciparum*. Cell Host Microbe *26*, 35–47.

Ruecker, A., Mathias, D.K., Straschil, U., Churcher, T.S., Dinglasan, R.R., Leroy, D., Sinden, R.E., and Delves, M.J. (2014). A male and female gametocyte functional viability assay to identify biologically relevant malaria transmission-blocking drugs. Antimicrob. Agents Chemother. *58*, 7292–7302.

Sastry, G.M., Adzhigirey, M., Day, T., Annabhimoju, R., and Sherman, W. (2013). Protein and ligand preparation: parameters, protocols, and influence on virtual screening enrichments. J. Comput. Aided Mol. Des. *27*, 221–234.

Siciliano, G., Santha Kumar, T.R., Bona, R., Camarda, G., Calabretta, M.M., Cevenini, L., Davioud-Charvet, E., Becker, K., Cara, A., Fidock, D.A., et al. (2017). A high susceptibility to redox imbalance of the transmissible stages of *Plasmodium falciparum* revealed with a luciferase-based mature gametocyte assay. Mol. Microbiol. *104*, 306–318.

Sridharan, S., Kurzawa, N., Werner, T., Gunthner, I., Helm, D., Huber, W., Bantscheff, M., and Savitski, M.M. (2019). Proteome-wide solubility and thermal stability profiling reveals distinct regulatory roles for ATP. Nat. Commun. *10*, 1155.

Taylor, H.M., McRobert, L., Grainger, M., Sicard, A., Dluzewski, A.R., Hopp, C.S., Holder, A.A., and Baker, D.A. (2010). The malaria parasite cyclic GMP-dependent protein kinase plays a central role in blood-stage schizogony. Eukaryot. Cell *9*, 37–45.

Tewari, R., Straschil, U., Bateman, A., Bohme, U., Cherevach, I., Gong, P., Pain, A., and Billker, O. (2010). The systematic functional analysis of *Plasmodium* protein kinases identifies essential regulators of mosquito transmission. Cell Host Microbe *8*, 377–387.

Urich, R., Wishart, G., Kiczun, M., Richters, A., Tidten-Luksch, N., Rauh, D., Sherborne, B., Wyatt, P.G., and Brenk, R. (2013). *De novo* design of protein kinase inhibitors by *in silico* identification of hinge region-binding fragments. ACS Chem. Biol. *8*, 1044–1052.

van der Pluijm, R.W., Imwong, M., Chau, N.H., Hoa, N.T., Thuy-Nhien, N.T., Thanh, N.V., Jittamala, P., Hanboonkunupakarn, B., Chutasmit, K., Saelow, C., et al. (2019). Determinants of dihydroartemisinin-piperaquine treatment failure in *Plasmodium falciparum* malaria in Cambodia, Thailand, and Vietnam: a prospective clinical, pharmacological, and genetic study. Lancet Infect. Dis. *19*, 952–961.

Van Voorhis, W.C., Adams, J.H., Adelfio, R., Ahyong, V., Akabas, M.H., Alano, P., Alday, A., Aleman Resto, Y., Alsibaee, A., Alzualde, A., et al. (2016). Open source drug discovery with the malaria box compound collection for neglected diseases and beyond. PLoS Pathog. *12*, e1005763.

Werner, T., Sweetman, G., Savitski, M.F., Mathieson, T., Bantscheff, M., and Savitski, M.M. (2014). Ion coalescence of neutron encoded TMT 10-plex reporter ions. Anal. Chem. *86*, 3594–3601.

WHO. (2019). World Malaria Report 2019 (World Health Organization). https://www.who.int/publications-detail/world-malaria-report-2019.

STAR★METHODS

KEY RESOURCES TABLE

REAGENT or RESOURCE	SOURCE	IDENTIFIER
Antibodies		
Cy3-conjugated anti-Pfs25 antibody	(Ruecker et al., 2014)	N/A
Mouse anti-HA antibody	Sigma-Aldrich	Cat# H3663; RRID: AB_262051
Rabbit anti-GAPDH antibody	Abcam	Cat# ab9485; RRID: AB_307275
Anti-mouse antibody	Thermo Fisher Scientific	Cat# 62-6520; RRID: AB_2533947
Anti-rabbit antibody	Cell Signaling Technology	Cat# 7074S; RRID: AB_2099233
Bacterial and Virus Strains		
Escherichia coli Rosetta2-pLysS cells	EMD Millipore	https://www.emdmillipore.com/
Biological Samples		
HEK-293 human embryonic kidney cell line	ATCC	https://www.atcc.org
K-562 human bone marrow cell line	ATCC	https://www.atcc.org
Hep G2 human hepatocellular carcinoma cell line	ATCC	https://www.atcc.org
Placenta (tissue protein extract)	ATCC	https://www.atcc.org
Chemicals, Peptides, and Recombinant Proteins		
All tested antimalarials and their structures are available in Figure S1.		N/A
MMV030084 (TCMDC-140369)	Medicines for Malaria Venture, Geneva, Switzerland	N/A
TCMDC-140680	GlaxoSmithKline, Tres Cantos, Spain	N/A
TCMDC-141334	GlaxoSmithKline, Tres Cantos, Spain	N/A
TCMDC-141060	GlaxoSmithKline, Tres Cantos, Spain	N/A
TCMDC-141070	GlaxoSmithKline, Tres Cantos, Spain	N/A
TCMDC-140700	GlaxoSmithKline, Tres Cantos, Spain	N/A
TCMDC-140762	GlaxoSmithKline, Tres Cantos, Spain	N/A
TCMDC-140549	GlaxoSmithKline, Tres Cantos, Spain	N/A
TCMDC-141154	GlaxoSmithKline, Tres Cantos, Spain	N/A
MMV030734 (TCMDC-141019)	Medicines for Malaria Venture, Geneva, Switzerland	N/A
BrightGlo reagent	Promega	https://www.promega.com/
MitoTracker Deep Red	Thermo Scientific	https://www.thermofisher.com/
D-Luciferin	PerkinElmer	https://www.perkinelmer.com/
TMTsixplex reagents	Thermo Scientific	https://www.thermofisher.com/
AKTA purifier	GE Healthcare	https://www.gehealthcare.com/
NHS-activated Sepharose matrix	Sigma-Aldrich	https://www.sigmaaldrich.com/
Mini-PROTEAN® TGX™ Precast Gels	Bio-Rad	https://www.bio-rad.com/
SuperSignal® West Pico Chemiluminescent substrate	Thermo Fisher Scientific	Cat# PI34080
anhydrotetracycline	Sigma-Aldrich	Cat# 37919
ML10	LifeArc	https://www.lifearc.org/
WR99210	Sigma-Aldrich	https://www.sigmaaldrich.com/
Critical Commercial Assays		
ADP-Glo Kinase Kit	Promega	https://www.promega.com/
Nextera XT kit	Illumina	https://www.illumina.com/

(*Continued on next page*)

Continued

REAGENT or RESOURCE	SOURCE	IDENTIFIER
Peptide substrate GRTGRRNSI-NH2	Sigma-Aldrich	Cat# SCP0212
Renilla-Glo(R) Luciferase Assay System	Promega	Cat# E2750
QiAmp DNA Blood Mini kit	Qiagen	https://www.qiagen.com/
Deposited Data		
PKG crystal structure	(Baker et al., 2017)	PDB ID: 5DYK
Experimental Models: Organisms/Strains		
Anopheles stephensi mosquitoes	Insectary Core Facility, New York University	N/A
Plasmodium falciparum asexual blood stage parasites	Goldberg lab, Washington University	3D7-A10 clone
P. falciparum asexual blood stage parasites	Fidock lab, Columbia University Medical Center	Dd2-B2 clone
P. falciparum NF54 CDPK1 T145M line	Miller lab, Malaria Cell Biology, NIAID, NIH (Bansal et al., 2018)	CDPK1^{T145M}
P. falciparum 3D7*elo1-pfs16*-CBG99 transgenic parasites	Fidock lab, Columbia University Medical Center (Cevenini et al., 2014)	N/A
BALB/c female mice	The Jackson Laboratory	https://www.jax.org/
P. berghei ANKA (Pb-Luc)	Insectary Core Facility, New York University	Pb-Luc, also known as *P. berghei*-ANKA-GFP-Luc-SMCON
Oligonucleotides		
All primers and cloning fragments are shown in Table S1 (J) and (K)		N/A
Recombinant DNA		
pSN054 vector	(Nasamu et al., 2019)	N/A
pET28-PfTKL3-SAM(V54E)-KD	(Abdi et al., 2010)	N/A
Software and Algorithms		
GraphPad Prism Version 8	GraphPad Software, San Diego, CA, USA	www.graphpad.com
ICY BioImage Analysis	(Delves et al., 2016)	http://icy.bioimageanalysis.org/
R Studio	RStudio Team, 2015	http://www.rstudio.com/
Proteome Discoverer	Thermo Scientific	https://www.thermofisher.com/us/en/home.html
Mascot	Matrix Science	http://www.matrixscience.com/
Scaffold Q+S	Proteome Software	http://www.proteomesoftware.com/
ChemiDoc™ MP System and Image Lab 5.2.0	Bio-Rad	https://www.bio-rad.com/
El- MAVEN software	(Agrawal et al., 2019)	https://elucidatainc.github.io/ElMaven/
Schrodinger molecular modeling suite	Schrodinger	http://www.schrodinger.com/

RESOURCE AVAILABILITY

Lead Contact

Further information and requests for resources and reagents should be directed to and will be fulfilled by the Lead Contact, David Fidock (df2260@cumc.columbia.edu).

Materials Availability

Please note that availability of experimental compounds may be restricted and might require resynthesis. Chemical structures for MMV030084 and select analogs from the Tres Cantos Antimalarial Set are shown in Figure S1A.

Data and Code Availability

All datasets generated during this study are provided in separate spreadsheets as part of Table S1.

No code was generated.

EXPERIMENTAL MODEL AND SUBJECT DETAILS

The *Pf* parasites used in this study were cultured in human O^+ blood (sex of donor unknown) at 3% hematocrit in RPMI-1640 media supplemented with 50 μM hypoxanthine, 2 g L^{-1} sodium bicarbonate, 2 mM L-glutamine, 25 mM HEPES, 0.5% AlbuMAXII (Invitrogen) and 10 μg mL^{-1} gentamycin in 5% O_2, 5% CO_2 and 90% N_2 at 37°C. The Dd2-B2 *Pf* line was cloned by limiting dilution from Dd2 (a kind gift from Thomas Wellems, NIAID, NIH, Rockville, MD). The 3D7-A10 *Pf* clone was provided by Dan Goldberg (Washington University, St. Louis, MO). The NF54 and CDPK1 conditional knockdown lines were provided by Louis Miller (NIAID, NIH, Rockville, MD) (Bansal et al., 2018). Routine sequencing of the drug resistance markers *pfcrt*, *dhfr* and *K13*, accompanied by whole-genome sequencing of parental lines, was used to authenticate cell lines.

METHOD DETAILS

Liver Stage Susceptibility Testing

P. berghei ANKA infections were maintained in BALB/c female mice (The Jackson Laboratory) infected via intraperitoneal injection of 2×10^7 blood stage parasites. Infected mice were provided water *ad libitum* and housed in standard cages in an animal facility room maintained at 22°C and 50-70% relative humidity. *Anopheles stephensi* mosquitoes were housed in an insectary maintained at 27°C, 80% humidity and a 12:12 light:dark schedule. The timing of MMV030084 activity against *P. berghei* liver stages was determined using a luciferase-based assay (Antonova-Koch et al., 2018). 24 h before infection, 384-well plates (Greiner Bio) were seeded with $1x10^4$ HepG2-A16-$CD81^{EGFP}$ cells in 30 μL of assay medium (DMEM without Phenol Red (Life Technologies, CA), 5% FBS and 5x Pen-Strep Glutamine (Life Technologies, CA)). Luciferase-expressing Pb (PbLuc) sporozoites were freshly obtained by dissecting salivary glands of infected *Anopheles stephensi* mosquitoes and $5x10^3$ sporozoites in 30 μL were added to each well. Plates were centrifuged for 5 min at 330g and incubated for 2 h at 37°C and 5% CO_2. Unbound sporozoites were then removed by centrifugation and washing and 30 μL of fresh medium was added. 12-point serial dilutions of test and control compounds in DMSO were added to the wells either 4 h before or 2 h after infection (corresponding to pre-infection and post-infection treatments, respectively). At 48 h post-infection, medium was removed and 20 μL of BrightGlo reagent (Promega) was added to each well. Luminescence was measured using an Envision Multilabel Reader (PerkinElmer) to assess parasite growth as a function of compound concentrations.

Asexual Blood Stage Susceptibility Testing

Unsynchronized parasites were exposed for 72 h to 10 different concentrations of MMV030084 or its analogs, plus control no-drug conditions, starting with predominantly rings (Murithi et al., 2019). We also exposed specific stages of highly-synchronized parasites to MMV030084 to determine the period of peak activity, defined as the asexual blood stage at which the compounds showed the lowest ${IC_{50}}^{8h}$ values (Murithi et al., 2019). Synchronicity was achieved using three rounds of sorbitol treatment on early rings, spaced 48 h apart, followed by magnetic purification of segmented schizonts following the final sorbitol treatment. Parasites were highly synchronous to within a 3 h window of development. Assays were repeated on at least three independent occasions (each with technical duplicates) and statistical significance was assessed using Mann Whitney *U* tests.

Gametocyte Susceptibility

This was determined using two separate assays. The first involved MitoTracker staining and high-content imaging. Mature (stage V) gametocytes were generated from NF54 ABS cultures as described elsewhere (Plouffe et al., 2016). Briefly, asexual parasites were cultured at 5% hematocrit in O^+ human erythrocytes in serum-containing complete media (RPMI 1640 (Life Technologies), 0.05 mg/ml gentamicin (Life Technologies), 0.014 mg/ml hypoxanthine (Sigma), 38.4 mM HEPES (Sigma), 0.2% (w/v) sodium bicarbonate (Sigma), D-glucose 0.2% (w/v) (Sigma), 3.4 mM sodium hydroxide (Sigma), 4.3% (v/v) heat-inactivated human serum (O^+, Interstate Blood bank), and 0.2% (w/v) AlbuMAX® II (Life Technologies)). Parasites were cultured at 37o̲C under low-oxygen conditions (3% O_2, 5% CO_2, 92% N_2) and parasitemias were maintained between 0.5% and 3% in cultures with 5% hematocrit. Ring-stage parasites underwent three rounds of synchronization with 5% (w/v) D-Sorbitol (Sigma), on days -8, -6 and -4 relative to the day of initiating gametocyte production. Trophozoite-stage parasites were shaken overnight at 37°C until day -1. On day -2 only 50% of the media was exchanged to provide additional stress to cultures, whose parasitemias had typically attained 7-10%. Complete media exchange was performed daily from day -1 onwards. Stage V gametocytes were treated with 50 mM N-acetyl glucosamine (Sigma) on days 0-9. Gametocytes were then diluted to 0.7% gametocytemia and 1.25% hematocrit in serum-free screening medium (RPMI 1640, 0.05 mg/ml gentamicin, 0.014 mg/ml hypoxanthine, 38.4 mM HEPES, 0.2% (w/v) sodium bicarbonate, 0.2% (w/v) D-glucose, 3.4 mM sodium hydroxide, and 0.4% (w/v) AlbuMAX II). Using a MultiFlo dispenser, 40 μl of culture was dispensed into 384-well plates pre-loaded with 50 nL of test or control compounds per well. Plates were then incubated at 37°C for 72 h under low O_2 conditions. Cells were stained by adding 10 μL of 2.5 μM MitoTracker Red CMXRos and 0.13% saponin solution in screening media to each well, followed by incubation for 120 min at room temperature in the dark. Plates were imaged using a high-content imaging system (Operetta, PerkinElmer) and Harmony software used for image analysis. The second assay assessed gametocyte susceptibility using a luciferase-based assay with 3D7*elo1-pfs16*-CBG99 transgenic parasites (Cevenini et al., 2014). Synchronized N-acetylglucosamine (NAG)-treated early and late stage gametocytes (day 4 and 11 of gametocyte development, respectively) were diluted to 2% hematocrit and exposed to eight serially diluted concentrations of MMV030084 for 48 h in 96-well plates (final volume of 100 μl/well). Cell viability was then evaluated by adding a non-lysing formulation of 0.5 mM D-Luciferin substrate and measuring

luciferase activity for 0.5 s on a Varioskan™ Flash Multimode Reader (Thermo Scientific). The percent of viability was calculated as a function of drug concentration and IC_{50} values were derived by non-linear regression analysis and curve fitting (using GraphPad Prism 6.0).

Dual Gamete Formation Assay

Pf NF54 gametocytes were generated as reported elsewhere (Delves et al., 2016). Briefly, static cultures of unsynchronized asexual parasites were continuously maintained as above. Parasites were cultured at 1% ring-stage parasitemias and 4% (v/v) hematocrit prior to the induction of gametocytes. To prepare gametocytes, 75% of the media was replaced daily for 14 subsequent days without adding fresh RBCs. During this process the temperature was kept strictly constant, as the rate of exflagellation is highly temperature-dependent. Between days 1 and 4 after culture induction, the asexual parasitemias increased rapidly to a peak. By day 7, as the asexual population waned, early-stage gametocytes became observable. By day 8, gametocyte maturation had progressed, and by day 14 morphologically distinguishable, mature stage V male and female gametocytes were present. A ten-point serial dilution of MMV030084 was prepared in DMSO and added to 100 μl culture medium in 1.5 ml tubes in a heater block at 37°C. Control reactions contained DMSO or 20 μM methylene blue (as negative and positive controls, respectively). Gametocyte cultures were diluted in culture medium to give 50 million cells/ml and then 100 μl was added to each 1.5 ml assay tube to give a final assay volume of 200 μl and 25 million cells/ml. Tubes were briefly gassed with 3% (vol/vol) O_2/5% CO_2/92% N_2, sealed and returned to a 37°C incubator for 24 h. For the carry-over protocol, gametogenesis was induced in the presence of the drug. For the wash-out protocol, gametogenesis was carried out only after cells had been washed three times to remove drug. Both the carry-over protocol tubes and the wash-out tubes were then treated identically. Cells were concentrated by removing 100 μl of supernatant and were then resuspended in the remaining 100 μl that was quickly transferred to wells of a flat-bottomed 96 well plate containing a 1:100 dilution of a Cy3-conjugated anti-Pfs25 antibody (Ruecker et al., 2014) in 10 μl ookinete medium to induce gametogenesis. The plate was then transferred to an automated wide-field microscope. Twenty min after induction, exflagellation centers were imaged with bright-field illumination using a 4x objective capturing 20 frames over 2 s. After imaging, the plate was incubated at 18°C for a further 24 h to allow female gametes to maximally express Pfs25, followed by fluorescence microscopy imaging using a 10× objective. Both time lapse images of exflagellation and fluorescence images of female gametes were automatically quantified using custom scripts in ICY Bio-Image Analysis (Delves et al., 2016). Inhibition was evaluated with respect to positive and negative controls (producing 100% or 0% inhibition, respectively).

Metabolomics

Metabolomics experiments were performed as described elsewhere (Murithi et al., 2019). Briefly, sorbitol-synchronized trophozoites were purified by MACS columns and either exposed to 10× the IC_{50} of MMV030084 for 3 h or allowed to develop to the schizont stage that was then exposed to 10× the MMV030084 IC_{50} for 3 h. Following several washes, metabolites were extracted by 90% methanol containing 0.5 μM $^{13}C^{15}N$-labelled aspartate as an internal standard. Nitrogen-dried samples were stored at -80°C. Samples were resuspended in high-performance liquid chromatography (HPLC) grade water that contained 1 μM chlorpropamide as an internal standard. They were then analyzed by ultra-high-performance liquid chromatography mass spectrometry UHPLC-MS (Allman et al., 2016). Data analysis was performed using the el-MAVEN software and the Metaboanalyst package in R Studio (Murithi et al., 2019).

Phosphoproteomics

Sorbitol-synchronized Dd2-B2 wild-type and ed. Dd2-B2 $TKL3^{KO}$ schizonts were exposed to 1200 nM (3× the Dd2-B2 wild-type IC_{50}) of MMV030084 or control conditions for 3 h. Red blood cells were lysed by 0.1% saponin in washing buffer (20 mM β-glycerophosphate, 1 tablet of cOmplete protease inhibitor cocktail (Roche), and 1 tablet of PhosSTOP phosphatase inhibitor (Roche) per 10 ml PBS). Parasites were subsequently washed three times in washing buffer. Parasite pellets were stored at -80°C until all samples from two independently repeated exposure experiments were collected. Proteins were extracted from schizont pellets by sonication and lysis in Tris-EDTA buffer (10 mM Tris-HCl, 5 mM EDTA, 20 mM ß-glycerolphosphate; pH 7.4, 1% NP-40, 2% CHAPS) followed by reduction of disulfide bonds and alkylation. Samples were digested with trypsin at a ratio of 1:15 (w/w) trypsin and proteins concentrated using S-trap columns (ProtiFi) for 1 h at 47°C. For tandem mass tag (TMT) labelling, digested samples were incubated with TMTsixplex reagents (Thermo Scientific). All labeled samples were pooled together, loaded onto an anion-exchange column, and fractionated on an AKTA purifier (GE Healthcare). The fractions were concentrated and phosphopeptides were enriched using titanium dioxide (TiO_2) and immobilized metal affinity chromatography (IMAC). LC-MS/MS was carried out using an Orbitrap Fusion with a RSLCnano System (Thermo Scientific). The raw data from the LC-MS/MS acquisition was processed using Proteome Discoverer (Thermo Scientific) and Mascot (Matrix Science) to search each file against the UniProtKB-Swissprot database with variable modifications set for oxidation (M) and phosphorylation (S/T/Y). Data were further processed in Scaffold Q+S (Proteome Software) and then exported to Microsoft Excel to calculate the difference (fold change) in the mean log_2 normalized intensities between control and MMV030084-treated parasites for each peptide. We conducted a consensus motif analysis of the modified sequences for all the significantly upregulated/downregulated peptides in order to identify phosphoproteins that were putative PfPKG substrates. We also analyzed the peptide sequences for patterns that conformed to the previously established PfPKG consensus motif K/RR/KxpS/pT and the PfPKA consensus motif K/RxxpS/pT (Francis et al., 2010).

Chemoproteomics

Human extracts were prepared as described (Bantscheff et al., 2011). Briefly, frozen cell pellets were homogenized in lysis buffer (50 mM Tris-HCl, 0.8% Igepal-CA630, 5% glycerol, 150 mM NaCl, 1.5 mM $MgCl_2$, 25 mM sodium fluoride, 1 mM sodium vanadate, 1 mM DTT, pH 7.5). One cOmplete EDTA-free protease inhibitor tablet (Roche) was added per 25 ml. Samples were dispersed using a Dounce homogenizer, rotated for 30 min at 4°C, and centrifuged at 20,000g for 10 min at 4°C. Supernatants were then recentrifuged at 145,000g for 1 h at 4°C. Protein concentrations were determined using a Bradford assay (BioRad). Aliquots were snap frozen in liquid nitrogen and stored at −80°C.

Pf protein extracts were prepared as described elsewhere (Paquet et al., 2017). Parasite cultures at the trophozoite stage were harvested, washed twice in RPMI 1640 medium (Gibco), and lysed by the addition of saponin to a final concentration of 0.1% for 5 min at room temperature. Free parasites were sedimented by centrifugation (3500 rpm for 10 min), washed three times with phosphate-buffered saline, and stored at −80°C. Parasites were resuspended in cell lysis buffer (50 mM tris-HCl (pH 7.5), 5% glycerol, 1.5 mM $MgCl_2$, 1.5 mM NaCl, 1 mM Na_3PO_4, 25 mM sodium fluoride, 0.8% NP-40, EDTA-free protease inhibitor cocktail tablets (Roche Diagnostics), and 1 mM DTT) and disrupted by sonication (Sonics Vibra-Cell) using two 30-s cycles on ice at half-amplitude. After centrifugation (140,000g for 1 h at 4°C), soluble proteins were recovered from the supernatants, and protein concentrations of each sample were estimated using the Lowry-based DC assay (Bio-Rad). Samples were stored at −80°C until use.

Kinobeads were prepared by immobilizing Bis-(III) indolyl-maleimide, purvalanol B, staurosporine and CZC8004, and the analogs of PD173955, sunitinib and vandetanib on NHS-activated Sepharose 4 beads (Amersham) (Bantscheff et al., 2007). MMV030084 or TCMDC-141154 were attached at a concentration of 1 mM to a NHS-activated Sepharose matrix via their primary amine moiety (Bantscheff et al., 2007). The chemoproteomic affinity-capturing experiments were performed as previously described (Bantscheff et al., 2011). Briefly, beads were washed and equilibrated in lysis buffer (50 mM Tris-HCl, pH 7.4, 0.4 % Igepal-CA630, 1.5 mM $MgCl_2$, 5 % Glycerol, 150 mM NaCl, 25 mM NaF, 1 mM Na_3VO_4, 1 mM DTT, and one cOmplete EDTA-free protease inhibitor tablet (Roche) per 25 mL). The beads were incubated at 4°C for 1 h with either 0.1 ml (0.3 mg) *Pf* blood stage extract or 0.5 mL (2.5 mg) human cell/tissue protein extract (a mixture of HEK-293, K-562, Hep G2 , and placental cells), which was pre-incubated with compound or DMSO (vehicle control). 10 samples were measured in parallel (TMT 10-plex (Werner et al., 2014)) to generate values for the affinity of the beads to the bound proteins (“depletion” values, 4 samples) and to generate IC_{50} values (6 samples) in a single experiment. Apparent dissociation constants were determined after accounting for protein depletion by the beads (Bantscheff et al., 2011). Beads were transferred to Filter plates (Durapore PVDF membrane, Merck Millipore), washed with lysis buffer, and eluted with SDS sample buffer.

Proteins were digested according to a modified single pot solid-phase sample preparation (SP3) protocol (Hughes et al., 2014). Peptides were labeled with isobaric mass tags (Thermo Fisher Scientific, Waltham, MA) using 10-plex TMT reagents, enabling relative quantification of 10 conditions in a single experiment (Werner et al., 2014). The labeling reactions and LC-MS/MS measurements using Q Exactive Orbitrap or Orbitrap Fusion Lumos mass spectrometers (Thermo Fisher Scientific) were performed as described elsewhere (Sridharan et al., 2019). Mascot 2.4 (Matrix Science, Boston, MA) was used for protein identification by using a 10 parts per million mass tolerance for peptide precursors and 20 mD (HCD) mass tolerance for fragment ions. To create the fasta file for mascot searching, all proteins corresponding to the taxonomy ‘*Plasmodium falciparum* (isolate 3D7)’ were downloaded from Uniprot (release 20170621) and supplemented with common contaminant protein sequences of bovine serum albumin, porcine trypsin and mouse, rat, sheep and dog keratins. To assess the false discovery rate (FDR), “decoy” proteins (reverse of all protein sequences) were created and added to the database, resulting in a database containing a total of 14266 protein sequences, with 50% forward and 50% reverse. For peptide and protein identification of human proteins see (Sridharan et al., 2019). Unless stated otherwise, we accepted protein identifications as follows: (i) For single-spectrum to sequence assignments, we required this assignment to be the best match and a minimum Mascot score of 31 and a 10× difference of this assignment over the next best assignment. Based on these criteria, the decoy search results indicated <1% false discovery rate (FDR). (ii) For multiple spectrum to sequence assignments and using the same parameters, the decoy search results indicated <0.1% FDR. Quantified proteins were required to contain at least 2 unique peptide matches. FDR for quantified proteins was <0.1%. Raw data tables for the chemoproteomics experiments can be found in Table S1 (E1-3).

Generation of Constructs and Parasite Transfections for cKD Lines

To study the interaction between the compounds and PKG (PF3D7_1436600), AAT (PF3D7_1231400), CDPK1 (PF3D7_0217500), TKL3 (PF3D7_1349300), PP1 (PF3D7_1414400), and URP (PF3D7_0808300), cKD lines were generated by fusing the transcripts with RNA aptamers for translation regulation with the TetR/DOZI system (Ganesan et al., 2016). The right homology regions (RHR) of each target gene were amplified by PCR, using the primers listed in Table S1 (A), and along with the left homology regions (LHR) were fused to the re-codonized 3’-end region of the gene. Single guide RNA fragments were synthesized using the BioXP™ 3200 System (SGI-DNA; Table S1 (A)) and were cloned into the pSN054 vector (Nasamu et al., 2019) to install the C-terminal epitope tags V5 and 2x-hemagglutinin (HA) followed by a 10x RNA aptamer array and the TetR-DOZI expression cassette. This cassette also contained Blasticidin S-deaminase for selection in the parasites and the reporter gene *Renilla luciferase* (*RLuc*). The 3’-end of each target was re-codonized to avoid further modifications of the edited locus in the parasite via removal of the PAM site. Donor vector generation was carried out via Gibson assembly, and the final construct sequence was confirmed by restriction digests and Sanger sequencing.

Transfection into parasites was carried out by preloading erythrocytes with linearized vectors as described previously (Deitsch et al., 2001). Briefly, 50 μg of purified plasmid DNA was mixed with human RBC and subjected to 8 square wave electroporation

pulses of 365 V for 1 ms each, separated by 0.1 s in a 0.2 cm cuvette. The plasmid DNA preloaded cells were inoculated with NF54 parasites expressing Cas9 and T7 RNA polymerase, maintained in 500 nM anhydrotetracycline (aTc, Sigma-Aldrich 37919), and drug selection with 2.5 μg/mL of Blasticidin (RPI Corp B12150-0.1) was initiated four days after transfection. Emergence of transfectants was monitored via Giemsa smears and RLuc measurements.

Verification of Conditional Knockdown (cKD) Strategy

Immunoblotting was carried out to confirm the regulation of target protein expression by the TetR-DOZI-aptamer system. Parasites were cultured with (50 -nM) or without aTc for 72 h, and protein extracts from saponinlysed cells were mixed with protein loading buffer, containing sodium dodecyl sulfate (SDS) and dithiothreitol (DTT), and loaded onto Mini-PROTEAN® TGX™ Precast Gels (4-15% gradient) in Tris-glycine buffer. Polyacrylamide gel electrophoresis separated proteins were transferred to a polyvinylidene fluoride membrane using a Mini Trans-Blot Electrophoretic Transfer Cell system according to the manufacturer's instructions and blocked with 100 mg/mL skim milk in 1×TBS/0.1% Tween. Membrane-bound proteins were probed with mouse anti-HA (1:3000; Sigma, H3663) and rabbit anti-GAPDH (1:5000; Abcam, AB9485) primary antibodies, and anti-mouse (1:5000; Thermo Fisher Scientific, 62-6520) and anti-rabbit (1:5000; Cell Signaling, 7074S) horseradish peroxidase (HRP)-conjugated secondary antibodies. Following incubation in SuperSignal® West Pico Chemiluminescent substrate (Thermo Fisher Scientific, PI34080), protein blots were imaged and analyzed using the ChemiDoc™ MP System and Image Lab 5.2.0 (Bio-Rad). The theoretical molecular weights of the fusion proteins are 60.8, 101.8, 39, and 31.6 kDa for CDPK1, PKG, PP1, and the putative ubiquitin regulatory protein (URP), respectively. Because no band was detected from AAT and TKL3 lysates following multiple experiments in both control and cKD experiments, diagnostic PCRs using the primers listed in Table S1 (J) were employed to verify that the machinery for cKD was in place.

Growth Assays Using cKD Lines

Parasite proliferation rates were assessed in the presence and absence of aTc, using luminescence as a readout of growth. Synchronous ring-stage parasites were set up in triplicate in 96-well U-bottom BD Falcon™ plates and cultured in the presence (50 nM) and absence of aTc. Expansion was measured at 0 and 72 h by quantifying luminescence using the Renilla-Glo(R) Luciferase Assay System (Promega E2750) and the GloMax® Discover Multimode Microplate Reader (Promega). Parasite growth was determined from normalized RLuc values, with samples treated with 200 nM chloroquine (a lethal concentration) included as no-growth controls. Results were processed and visualized using Prism 8 (GraphPad Software). Experiments were performed on two independent occasions and statistical significance was assessed using an unpaired *t* test.

Compound Susceptibility Assays Using cKD Lines

Stock solutions of MMV030084, MMV030734, and TMDC-140549 were dispensed into 96-well U-bottom BD Falcon™ plates and serially diluted in complete medium to yield final concentrations ranging from 0.8-200 nM for MMV030084 and MMV030734 and 2.3-600 nM for TMDC-140549. Synchronized ring-stage cKD parasites in varying aTc concentrations (high = 50 nM, intermediate = 1.5 nM, low = 1 nM, and no aTc) were distributed into the drug plate. No-drug treatment and treatment with a lethal dose of chloroquine (200 nM) served as reference controls. Growth inhibition at the different concentrations was analyzed after 72 h using the luciferase assay described above, and EC_{50} values were obtained from dose-response curves using Prism 8 (GraphPad Software).

PfPKG Docking Studies

The PfPKG structure was downloaded from the Protein Data Bank (PDB 5DYK). All calculations were performed using the Schrodinger molecular modeling suite (version 2018-2). This structure was analyzed using the Protein Preparation Wizard where force field atom types and bond orders were assigned, missing atoms including hydrogens were added, tautomer/ionization states were assigned, residues were flipped to optimize the hydrogen bond network, and a constrained energy minimization was performed (Sastry et al., 2013). The Receptor Grid Generator tool was employed to create a glide grid of 30 x 30 x 30 $Å^3$ around the predicted drug-binding site. The compounds for docking were imported into Maestro as MDL SD files and prepared using the LigPrep tool at pH 7. Compounds were docked into the PfPKG glide grid using GlideDock_XP (extra precision) with a van der Waals radii of ligand atoms scaling factor of 0.80 and a partial charge cutoff for polarity of 0.15, followed by post-docking minimization (Friesner et al., 2004). Poses and H-bonding interactions were displayed using the XP Visualizer.

Recombinant PfPKG Inhibition Assay

Full length PfPKG (PF3D7_1436600) was expressed in *Escherichia coli* as previously described (Baker et al., 2017). The N-terminal His-tagged recombinant PfPKG protein was purified using HisTrap HP columns (GE Healthcare), followed by anion exchange and size exclusion chromatography (HiLoad 16/600 Superdex 200 pg column, GE Healthcare). Final buffer composition of purified protein was 50mM Tris-HCL pH 8.0, 150 mM NaCl, 10 mM β-mercaptoethanol, and 10% glycerol.

Kinase inhibition assays were preformed based on previously described methods (Baker et al., 2017; Penzo et al., 2019), using ADP formation as a measure of kinase activity. Briefly, 3-fold serial dilutions of MMV030084 were prepared in assay buffer (25 mM HEPES pH 7.4, 0.01 % (w/v) BSA, 0.01 % (v/v) Triton-X 100, 20 mM $MgCl_2$, 2 mM DTT, 10 μM cGMP). The final kinase reaction contained 1 nM purified PfPKG protein, 10 μM ATP (~ ½ the K_m of the enzyme), 20 μM peptide substrate GRTGRRNSI-NH2 (Sigma-Aldrich, Cat no. SCP0212), 1 % (v/v) DMSO and 1 μM – 0.15 nM inhibitor in assay buffer. Reactions were initiated by the addition of PfPKG and

incubated for 30 minutes at 22°C (resulting in <10% ATP conversion). ADP formation was measured using the ADP-Glo Kinase Kit (Promega) according to manufacturer's instructions. Briefly, 4 μl ADP-Glo Reagent was added to 4 μl kinase reaction in white 384 shallow-well plates (Nunc, Cat no. 264706) and incubated for 40 minutes at 22°C to deplete the remaining ATP. 8 μl of Kinase Detection Reagent was then added and the reaction was incubated for a further 30 minutes at 22°C. Luminescent signal was measured using the EnSpire Multimode Plate Reader (PerkinElmer). Data were normalized based on the DMSO controls (no inhibitor, 100% activity) and the 100% inhibition controls (1μM PfPKG inhibitor ML10 (Baker et al., 2017), LifeArc). The 100% inhibition controls gave comparable readings to the negative controls (no enzyme). Reactions were carried out in duplicate and IC_{50} values were calculated based on the average of three independent experiments using GraphPad Prism (log(inhibitor) vs. normalized response – Variable slope).

Selections for MMV030084 Resistance

Selections were initiated by exposing two independent flasks each containing 10^9 wild-type Dd2-B2 parasites to 450 nM of MMV030084, i.e. 3× the 72 h ABS IC_{50}. Medium containing MMV030084 was refreshed daily until sensitive parasites were effectively cleared, after which the medium was refreshed every other day and the culture volume was reduced gradually (as described elsewhere (Istvan et al., 2019)). The resulting MMV030084-resistant parasites were cloned by limiting dilution (Baker et al., 2017) to obtain genetically homogenous cultures for further phenotyping and whole-genome sequencing. A second round of selections using the ed. Dd2-B2 $TKL3^{KO}$ line included continuous exposure selections, as described above, and a pulsing selection in which parasites were exposed to low drug concentrations (starting at 525 nM MMV030084) for shorter intervals and allowed to recover before being exposed to gradually higher drug concentrations (up to 875 nM over the course of 67 days). Drug susceptibility of resistant lines was assessed using a 72 h ABS assay as described above, and a Mann Whitney *U* test was applied to assess statistically significant differences.

Whole-Genome Sequencing

DNA was extracted using the QiAmp DNA Blood Mini kit (Qiagen) and subjected to whole-genome sequencing using the Illumina TruSeq DNA PCR-Free library preparation protocol and a MiSeq (Illumina) sequencing platform. Briefly, 2 μg of genomic DNA were sheared to 550 bp size, end-repaired, adenylated on 3' ends and ligated with adaptors. The samples were pooled and sequenced on an Illumina MiSeq flow cell to obtain 300 bp paired end reads. The sequence data was aligned to the *Pf* parental genome (PlasmoDB version 36) using BWA (Burrow-Wheeler Alignment). Reads that did not map to the reference genome and PCR duplicates were removed using Samtools and Picard. The reads were realigned around indels using GATK RealignerTargetCreator and base quality scores were recalibrated using GATK Table-Recalibration. GATK HaplotypeCaller (version 3.8) (Min Base quality score $\geq$ 17) was used to identify of all possible variants in clones which were filtered based on quality scores (variant quality as function of depth QD > 1.5, mapping quality > 40), read depth (depth of read > 5) to obtain high-quality single nucleotide polymorphisms that were annotated using snpEFF. The list of variants from resistant clones were compared against the Dd2-B2 parental clone to obtain homozygous single nucleotide polymorphisms present exclusively in the resistant clones. Integrated Genome Viewer was used to confirm polymorphisms present in resistant clones.

CRISPR/Cas9 Editing of TKL3

A CRISPR/Cas9 targeted gene editing strategy using an all-in-one plasmid approach was applied to introduce T1268R, I1250M and T1268R+I1250M in wild-type Dd2-B2 parasites or to produce a TKL3 KO line by inserting a stop codon and frameshift at amino acid position 1268. This all-in-one plasmid encoded (i) a codon-optimized Cas9 nuclease, (ii) a single guide RNA (5'-GATATTTGCTAGAA GATTAAG-3' or 5'-GTGTCTATCATCATGCGTTTC-3') to complex the Cas9 nuclease to the target sequence, (iii) a *tkl3* donor sequence (nucleotides 3649–4019 of the PF3D7_1349300 coding sequence) that contained T1268R and/or I1250M, or T1268* and 10 binding site mutations to prevent Cas9 cleavage of the edited locus through any of the two guides, and (iv) a human *dhfr* cassette that mediates resistance to WR99210. Sorbitol-synchronized ring-stage cultures were transfected with two such plasmids each containing a different guide RNA sequence to maximize the chance of successful editing. Transfections were performed with a Gene Pulser II (Bio-Rad) at 0.31 kV and 910 μF at maximum capacitance. WR99210 (2.5 nM) was added to the culture medium 24 h after electroporation to select for successfully transfected parasites and was maintained in the medium until edited parasites were detected. Genetic editing was verified by PCR and Sanger sequencing and edited lines were subjected to limiting dilution cloning to obtain genetically homogeneous parasite populations.

Expression, Purification and Kinase Assay of WT and Kinase Dead Recombinant *Pf*TKL3 Kinase Domain

Recombinant proteins were expressed in *E. coli* as previously described (Abdi et al., 2010). Briefly, *E. coli* Rosetta2-pLysS cells (Novagen), transformed with expression plasmids pET28-PfTKL3-SAM(V54E)-KD expressing the wild-type or catalytically inactive kinase domain of TKL3, were inoculated 1:500 into 500 ml 2YT culture medium with kanamycin/chloramphenicol (100 and 34 mg/ml, respectively) at 37°C, with agitation at 200 rpm, and cultured up to an OD_{600} of 0.5. The cultures were then cooled to 20°C followed by induction of protein expression with 0.5 mM IPTG and further incubation at 20°C, 200 rpm for 16 h. Cultures were harvested by centrifugation at 4000g, 4°C for 10 min. Bacterial pellets were washed once in 1× PBS and were stored at −80°C. Cell pellets were resuspended in ice-cold Lysis Buffer (50 mM sodium phosphate, pH 8.0, 400 mM NaCl, 5 % (w/v) glycerol, 0.1 % (v/v) Triton X-100, 20 mM imidazole, 2 mM β-mercaptoethanol, 1× Roche protease inhibitor cocktail EDTA-free, 1 mg/ml lysozyme). Cells were

disrupted by sonication. Protein was bound on a HisTrap HP column and eluted with a linear gradient of increasing imidazole concentrations. The peak fractions as determined by A280 trace were pooled together and further purified by size exclusion chromatography on a S200 gel filtration column equilibrated in 25 mM Tris pH 7.5, 100 mM NaCl, 5 % (w/v) glycerol, 1 mM DTT. Protein was pooled and concentrated. Final concentration was determined using a Bradford assay with a BSA protein standard.

Kinase assays containing 3 μg of recombinant kinase (WT or mutant) were performed in kinase assay buffer (25 mM Hepes, pH 7.5, 2 mM $MnCl_2$, 10 mM β-glycerophosphate, 10 mM NaF, 25 μM ATP (containing 5 μCi [γ-^{32}P]-ATP-3000 mCi/mmol (PerkinElmer)), 5 μg substrate myelin basic protein (MBP) and ± 20 μM inhibitor) in a final reaction volume of 20 μl and incubated at 30°C for 30 min. Reactions were stopped by the addition of 5× Laemmli sample buffer and heated to 98°C for 5 min. Proteins were resolved by 4-12 % SDS-PAGE and stained in Coomassie Brilliant Blue. Kinase activity was detected by phosphorimaging. Imaging was performed on a Typhoon 5 Biomolecular imager (GE Healthcare Life Sciences).

QUANTIFICATION AND STATISTICAL ANALYSIS

All biochemical curves and statistical analyses were produced using Prism 8 (GraphPad Software). Details regarding statistical tests are reported in the Figure Legends and Supplemental Information.

Cell Chemical Biology, Volume *27*

Supplemental Information

Inhibition of Resistance-Refractory *P. falciparum* Kinase PKG Delivers Prophylactic, Blood Stage, and Transmission-Blocking Antiplasmodial Activity

Manu Vanaerschot, James M. Murithi, Charisse Flerida A. Pasaje, Sonja Ghidelli-Disse, Louis Dwomoh, Megan Bird, Natasha Spottiswoode, Nimisha Mittal, Lauren B. Arendse, Edward S. Owen, Kathryn J. Wicht, Giulia Siciliano, Markus Bösche, Tomas Yeo, T.R. Santha Kumar, Sachel Mok, Emma F. Carpenter, Marla J. Giddins, Olalla Sanz, Sabine Ottilie, Pietro Alano, Kelly Chibale, Manuel Llinás, Anne-Catrin Uhlemann, Michael Delves, Andrew B. Tobin, Christian Doerig, Elizabeth A. Winzeler, Marcus C.S. Lee, Jacquin C. Niles, and David A. Fidock

Inhibition of the resistance-refractory *P. falciparum* kinase PKG delivers prophylactic, blood stage and transmission-blocking antiplasmodial activity

Manu Vanaerschot, James M. Murithi, Charisse F.A. Pasaje, Sonja Ghidelli-Disse, Louis Dwomoh, Megan Bird, Natasha Spottiswoode, Nimisha Mittal, Lauren B. Arendse, Edward S. Owen, Kathryn J. Wicht, Giulia Siciliano, Markus Bösche, Tomas Yeo, T.R. Santha Kumar, Sachel Mok, Emma F. Carpenter, Marla J. Giddins, Olalla Sanz, Sabine Ottilie, Pietro Alano, Kelly Chibale, Manuel Llinás, Anne-Catrin Uhlemann, Michael Delves, Andrew B. Tobin, Christian Doerig, Elizabeth A. Winzeler, Marcus C.S. Lee, Jacquin C. Niles, David A. Fidock

Supplementary Figures and Tables (Table S1 is available as a separate multi-tab Excel file)

**Figure S1.**	Structure of the MMV030084 analogs tested in this study.	**p. 2**
**Figure S2.**	Female gamete formation assay results, showing little to no activity against female gametes.	**p. 3**
**Figure S3.**	Immunoblotting confirmed knock-downs of PKG, CDPK1, PP1 and URP.	**p. 4**
**Figure S4.**	Phosphorimage of recombinant TKL3 kinase inhibition studies indicate that MMV030084 and the tested analogs do not directly inhibit TKL3 activity.	**p. 5**
**Table S1 (A).**	MMV030084 asexual blood stage activity data.	
**Table S1 (B).**	Activity (IC_{50} in nM) of MMV030084 and its analogs against Dd2-B2 and a TKL3-mutant line.	
**Table S1 (C).**	Log_2 fold changes in metabolite levels induced by MMV030084 treatment of trophozoites and schizonts.	
**Table S1 (D1,2).**	Phosphorylated peptide levels in MMV030084-treated vs untreated (control) Dd2-B2 (D1) and TKL-3 KO (D2) parasites.	
**Table S1 (E1-3).**	Results from the chemoproteomic experiments.	
**Table S1 (F).**	Effect of cKDs on parasite growth.	
**Table S1 (G).**	Susceptibility data for the cKD lines to MMV030084 and MMV030734.	
**Table S1 (H)**.	MMV030084 and its analogs dock well into the crystal structure of PKG.	
**Table S1 (I).**	IC_{50} data for MMV030084-resistant lines.	
**Table S1 (J).**	Sequences of primers used to generate and validate cKD lines.	
**Table S1 (K).**	Homology regions and sgRNA fragments used to generate cKD lines.	

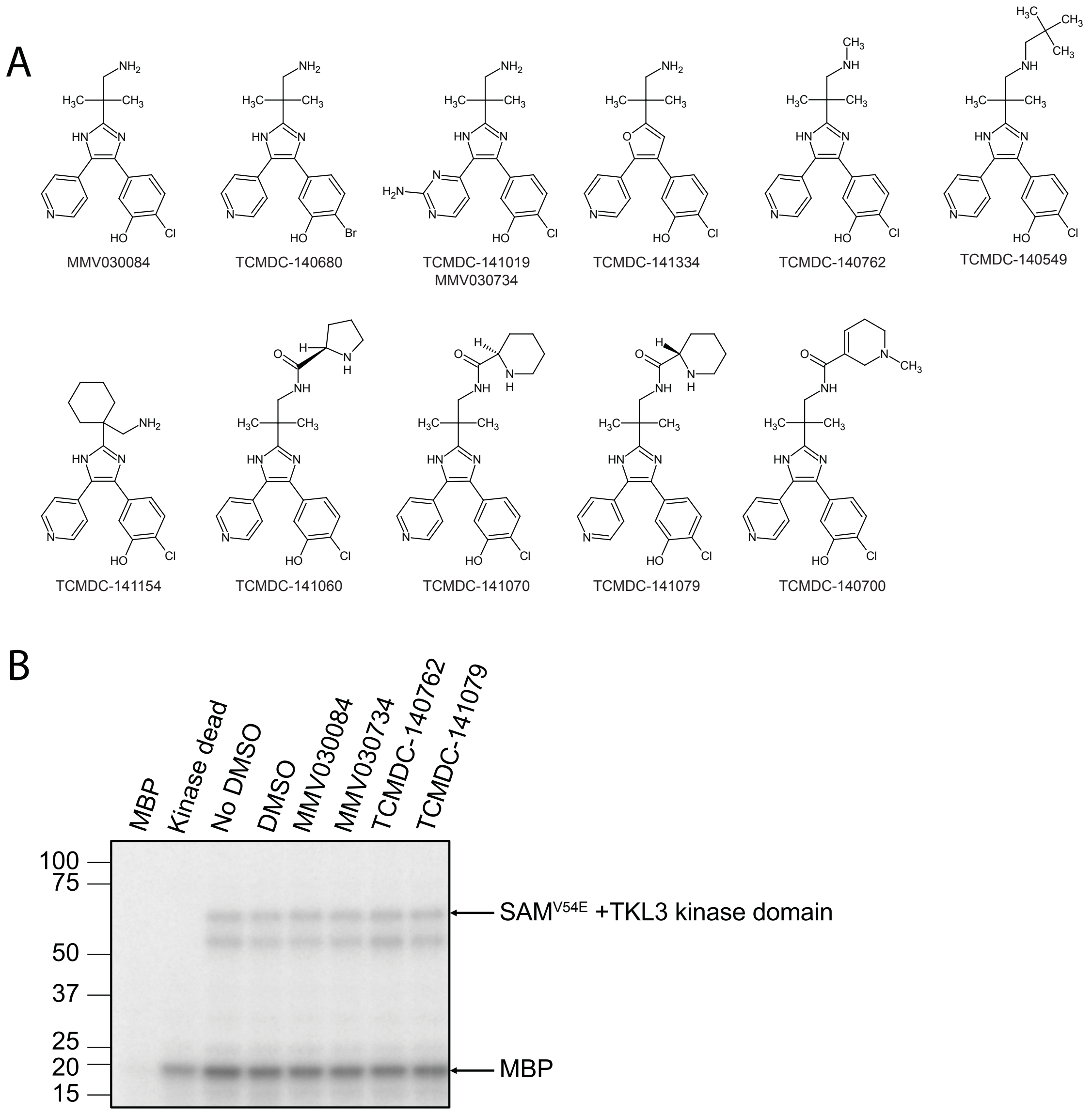

Figure S1. Structure of MMV030084 and its analogs and evidence of their lack of direct inhibition of TKL3 kinase activity (related to Figure 1 and Figure 3). (A) Analogs of MMV0300084. (B) Phosphorimage of recombinant TKL3 kinase inhibition studies indicate that MMV030084 and the tested analogs do not directly inhibit TKL3 activity. MBP: myelin basic product, SAM: sterile alpha motif.

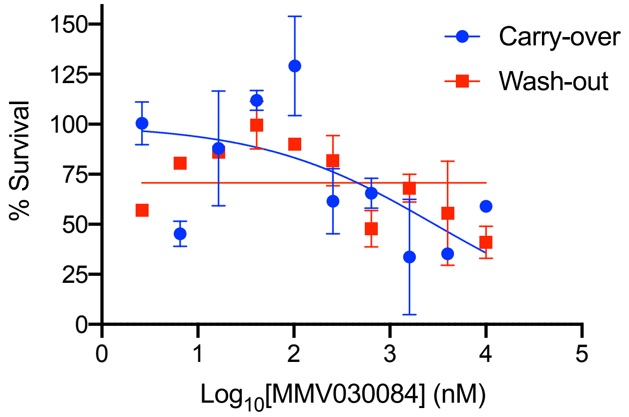

Figure S2. Female gamete formation assay results, showing little to no activity against female gametes (related to Figure 1). Error bars represent the standard error of the mean based on two independently repeated experiments.

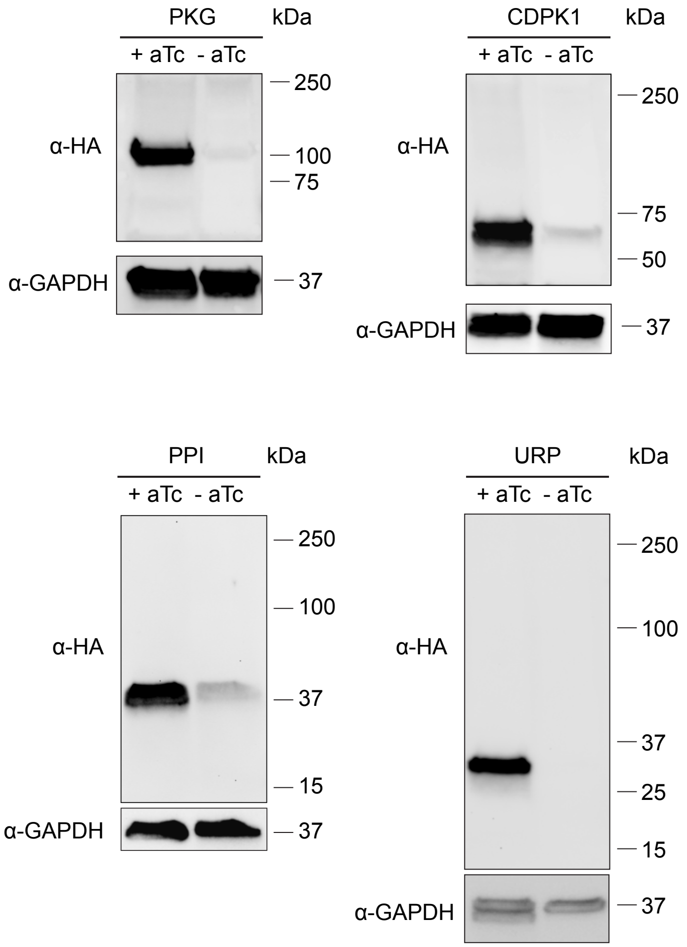

Figure S3. Immunoblotting confirmed knock-downs of PKG, CDPK1, PP1 and URP (related to Figure 3; Table S6). Proteins were probed using anti-HA antibodies, which target the HA tag that was introduced at the 3' end of the genes together with the cKD machinery**.**
